# Transcriptomic Changes Associated with Loss of Cell Viability Induced by Oxysterol Treatment of a Retinal Photoreceptor-Derived Cell Line: An In Vitro Model of Smith–Lemli–Opitz Syndrome

**DOI:** 10.3390/ijms22052339

**Published:** 2021-02-26

**Authors:** Bruce A. Pfeffer, Libin Xu, Steven J. Fliesler

**Affiliations:** 1 Department of Ophthalmology (Ross Eye Institute), Jacobs School of Medicine and Biomedical Sciences, SUNY-University at Buffalo, Buffalo, NY 14209, USA; brucepfe@buffalo.edu; 2 Research Service, VA Western NY Healthcare System, Buffalo, NY 14215, USA; 3 Department of Medicinal Chemistry, School of Pharmacy, University of Washington, Seattle, WA 98195, USA; libinxu@uw.edu; 4 Departments of Biochemistry and Neuroscience Program, Jacobs School of Medicine & Biomedical Sciences, SUNY-University at Buffalo, Buffalo, NY 14203, USA

**Keywords:** Smith–Lemli–Opitz, oxysterol, cholesterol, photoreceptor, gene array, cell stress, cell death, neurodegeneration

## Abstract

Smith–Lemli–Opitz Syndrome (SLOS) results from mutations in the gene encoding the enzyme DHCR7, which catalyzes conversion of 7-dehydrocholesterol (7DHC) to cholesterol (CHOL). Rats treated with a DHCR7 inhibitor serve as a SLOS animal model, and exhibit progressive photoreceptor-specific cell death, with accumulation of 7DHC and oxidized sterols. To understand the basis of this cell type specificity, we performed transcriptomic analyses on a photoreceptor-derived cell line (661W), treating cells with two 7DHC-derived oxysterols, which accumulate in tissues and bodily fluids of SLOS patients and in the rat SLOS model, as well as with CHOL (negative control), and evaluated differentially expressed genes (DEGs) for each treatment. Gene enrichment analysis and compilation of DEG sets indicated that endoplasmic reticulum stress, oxidative stress, DNA damage and repair, and autophagy were all highly up-regulated pathways in oxysterol-treated cells. Detailed analysis indicated that the two oxysterols exert their effects via different molecular mechanisms. Changes in expression of key genes in highlighted pathways (*Hmox1*, *Ddit3*, *Trib3*, and *Herpud1*) were validated by immunofluorescence confocal microscopy. The results extend our understanding of the pathobiology of retinal degeneration and SLOS, identifying potential new druggable targets for therapeutic intervention into these and other related orphan diseases.

## 1. Introduction

Smith–Lemli–Opitz Syndrome (SLOS) is caused by an inherited, autosomal recessive genetic defect targeting the final step in the cholesterol (CHOL) synthesis pathway, specifically affecting the gene encoding the enzyme 7-dehydrocholesterol reductase [DHCR7; EC 1.3.1.21], which catalyzes this biochemical step [1,2,3]. The severity of SLOS in human patients is governed by the precise loci of any of scores of mutations affecting either or both of the *DHCR7* alleles, which may lead to expressed protein with residual enzymatic activity, or to complete lack of functional gene product [4,5]. The resulting phenotypes can range from embryonic lethality to physical and cognitive impairments, some extremely profound, and which ultimately can result in death within the first few decades of life [6,7]. Some manifestations of the pathophysiology of this disease are certainly due to the reduced production of CHOL, and of its supply not meeting specific needs at the cellular, organ, and system level, with repercussions for cell membrane structure and function, as well as for endocrine and cellular signaling pathways [8,9]. In addition, it has become increasingly apparent that a significant etiologic factor in SLOS stems from the accumulation of 7-dehydrocholesterol (7DHC), the immediate precursor of CHOL [10]. This correlation with 7DHC may not be fully attributable to the inherent properties of 7DHC itself, although its substitution for CHOL can modulate the structure and function of cell membranes [11], and 7DHC has been shown to dysregulate Wnt/β-catenin signaling pathways [12]. More likely it is a result of the fact that 7DHC is extraordinarily prone to oxidation [13], generating a host of oxidatively modified sterols (oxysterols). Such molecules have been shown to exhibit potent effects at the cellular level, inducing selected gene expression changes, altered morphology, and loss of viability, resulting in cell death, at concentrations in the low micromolar range when assessed using neural cells in in vitro assay systems [14]. Many, if not most, of the oxysterol by-products of 7DHC have been isolated from tissues and bodily fluid obtained from SLOS patients [15].

A viable animal model of SLOS has been developed by treating rats with a small molecule inhibitor of DHCR7 (AY9944), beginning in utero and up to 3 postnatal months (depending on variable survival of the subjects) [16]. Notably, this rat SLOS model exhibits progressive and lamina-specific degeneration and dropout of retinal photoreceptor cells, beginning just after one postnatal month [16]. This morphological phenotype was found to correlate with electrophysiologic abnormalities, and was also linked to distinct changes in gene and protein expression in vivo, as well as alterations of proteomic, lipidomic, and metabolomic profiles in the neural retina [10,17,18,19]. Importantly, the analysis of sterols from the retinas of the rat SLOS model confirmed the formation and accumulation of several 7DHC-derived oxysterols as detected in tissues and plasma from human SLOS patients as well as in genetically altered animal models of this disease [20]. Taking into account all retinal neuronal and glial cell types, and also the retinal pigment epithelium (RPE) in the rat SLOS model, only the photoreceptors were shown to lose viability, as assessed by quantitative morphometric analysis and by TUNEL assay, in a reproducible fashion, suggesting that photoreceptors were preferentially susceptible to the cytotoxic effects of one or more 7DHC-derived oxysterols [10,16,19].

In vitro methods were implemented subsequently by our laboratory to evaluate the differential effects of exposure of pure cell cultures, representing photoreceptor cells, Müller glial cells, or RPE cells, to 7DHC-derived oxysterols—including 7-ketocholesterol (7kCHOL) and 5,9-endoperoxy-cholest-7-en-3β,6α-diol (EPCD), the latter being unique to the SLOS phenotype—as well as to CHOL itself [21,22]. Using a series of quantitative cell viability assays, we confirmed that the cytotoxic sensitivity to these compounds observed in 661W cells (a transformed mouse cone photoreceptor-derived cell line) was an order of magnitude greater than that observed for either rMC-1 cells (a transformed rat Müller cell-derived line), or normal diploid RPE cells originally isolated from rhesus macaque [21]. In addition, while both 7kCHOL and EPCD exposure caused complete cell death at the highest concentrations tested, EPCD was found in these studies to be 10- to 100-fold more potent than 7kCHOL (depending on the assay and physical growth parameters), with respect to dose–response kinetics affecting loss of cell viability for 661W cells [21].

Here we report the results of a gene array study designed to further discover, validate—and, ideally, predict—mechanistic aspects of oxysterol-induced cell death and dysfunction, using 661W cells as a tractable surrogate in vitro model system. An additional purpose was to discern differences in gene expression responses induced by 7kCHOL vs. the SLOS-specific oxysterol EPCD, as revealed by the comparative transcriptomic profiles induced by these two distinct molecules, while also accounting for changes brought about by incubation with the native, non-toxic control sterol CHOL. By means of our study design and analytical methods, we also intended to learn more in general regarding photoreceptor cell death and survival pathways in diseases of, and resulting from environmental damage to, the retina, and potentially to extend our conclusions to other central nervous system (CNS) cells and tissues. We exploited the array results to elucidate some previously unknown differentially expressed genes (DEGs), revealing novel correlations with the pathophysiology of retinal degenerations and/or SLOS, as neural degenerative conditions. Many of the changes in gene expression were expected, because they were emblematic of known signaling pathways involved in cell death, and also in cell survival. Gene ontology and enrichment results also reflected upstream and downstream cellular functions and processes connecting such mechanisms with, first, the primary changes (e.g., oxidative stress and DNA damage) brought about by the experimental agents, and then, the eventual cellular effectors of responses to stress. One general finding was that multiple modes of cell death could be documented based on gene expression patterns in oxysterol-treated cells, permitting the exploration of commonalities between our model, and cell death and survival pathways already ascribed to photoreceptors and other neuronal cell types. Finally, we validated the correlation of transcription, and translation to protein, of selected signature DEGs that were identified in this study, by immunofluorescent detection of changes in expression of proteins associated with these stress responses.

## 2. Results

### 2.1. Validation of 661W Cell Culture Response to Treatments

#### 2.1.1. Isolation of RNA: Rationale

**Figure 1:**
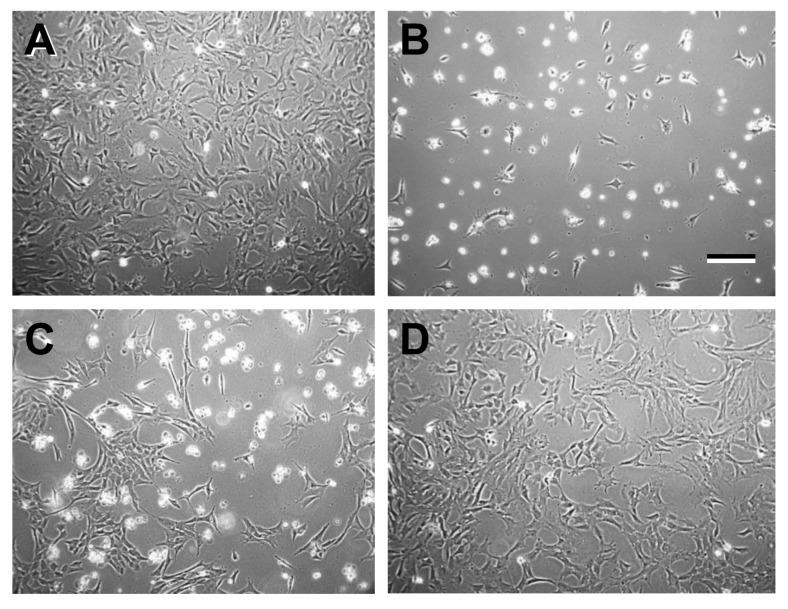
Phase-contrast micrographs recorded from 661W cells treated with incubation media containing (**A**) vehicle control (VC; hydroxypropyl-β-cyclodextrin) for 24 h; (**B**) 6 μM EPCD for 23 h; (**C**) 16 μM 7kCHOL for 5 h; and (**D**) 8 μM CHOL for 23 h, corresponding to the times of harvesting of total RNA from parallel triplicate samples for gene array analysis. Scale bar (panel B, for all panels): 200 μm.

Previous viability studies of the effects of EPCD and 7kCHOL (see Supplementary Materials, Figure S1 for structures) on 661W cells documented that toxic concentrations of these oxysterols brought about complete cell death within 24 h of incubation, and assay measurements (using a 48-well plate format) at earlier time points demonstrated that significant loss of viability occurred within the first 6 h of exposure to these agents [21]. Pilot experiments, in which we visually monitored the progress of the cells in response to challenge by oxysterols, were designed to reveal the optimal doses, exposure times, and formats (i.e., “growth” area) for either gene array or validating immunofluorescence experiments. Our overall reasoning was that we would obtain the most informative results by isolating RNA, and by detecting protein immunoreactivity as well, from individual cultures in which we documented a heterogeneous distribution of cellular morphologies, ranging from “normal” appearance, through evidence of retraction of normally observed neurites and loss of multipolarity, leading to rounding up of cells that maintained attachment and phase-refractility, to noticeable membrane deformities, culminating with only a small proportion finally in detachment and lysis. We utilized 100-mm culture dishes for gene array studies to maximize the amount of RNA in each sample, and found that EPCD- and 7kCHOL- treated cultures displayed different kinetics with respect to this range of morphological changes that were interpreted as indicative of progression towards universal cell death. As indicated in Figure 1, rounding up and loss of cells were more acute in 7kCHOL-treated dishes, and these cultures were processed for RNA isolation at 5 h, using a concentration of 16 µM 7kCHOL, a dose that we confirmed in parallel dishes ultimately led to virtually complete cell death over the full growth area by 24 h (results not shown). In contrast, the development of overt cell death in cultures exposed to 6 µM EPCD was less abrupt and more morphologically diverse (but also indicative of the higher potency of this latter oxysterol) (Figure 1); therefore, harvesting of RNA from EPCD-treated 661W cells at 23 h was deemed optimal, subsequent to the eventual cell death observed in separate dishes incubated under parallel conditions. At the same time, CHOL- and vehicle control (VC)-treated cultures at 23 and 24 h, respectively, showed no hallmarks of diminished viability (Figure 1); these cultures continued to demonstrate homogeneous, normal cell morphology and increased cell density compared to the experimental incubation start time.

#### 2.1.2. Principal Component Analysis (PCA)

**Figure 2:**
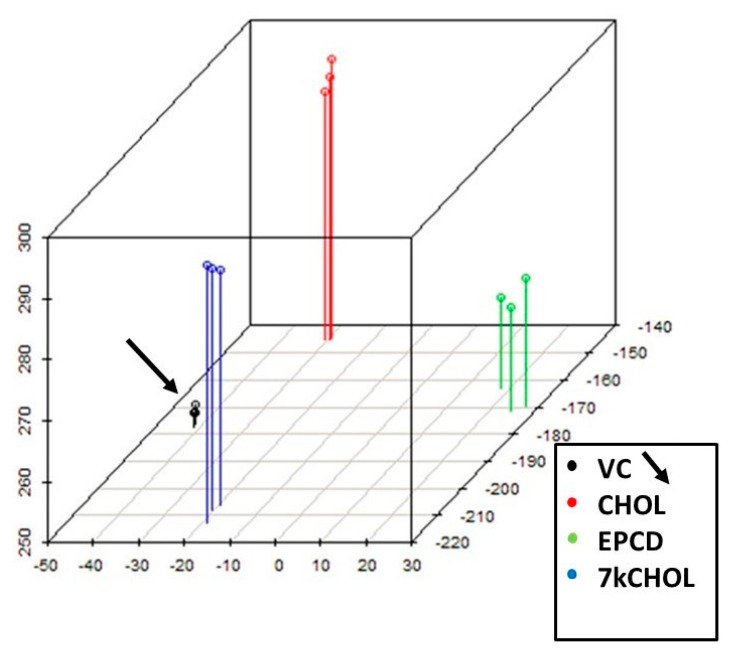
Principal component analysis for each sample treatment. Scatterplot of the triplicate samples for the individual array data, along the first three *principal components*, covering 89% of the total variability. The clustering of results for each individual sample reflects the similarities within each treatment group, which included vehicle control (VC, and *arrow*). The spatial separation of the data point clusters also suggests the distinct biological responses to each experimental treatment.

Present probe set data across all samples were visualized by means of principal components, employing a linear model [23] that fit and contrasted triplicate measurements from the four experimental conditions (i.e., including VC). PCA allowed the projection of the multivariate data vectors for each array across the first three principal components (*x*-, *y*-, and *z*-axes), covering 89% of the total variability of the samples (Figure 2). The spatial arrangement of the scatter plot reflects overall data similarity/dissimilarity between arrays, and in Figure 2, it is apparent that the four clusters of data points representing the three replicates for individual experimental reagent treatments each occupy different domains in three-dimensional space. In isolating independent components with the highest degree of variation [24], the spatial segregation seen in the PCA results also underscored the distinct biological responses to the separate experimental treatments, whether the final cell conditions were emblematic of cell survival (VC, CHOL) or demise (EPCD, 7kCHOL).

#### 2.1.3. Identification of DEGs for Each Treatment vs. VC

Differentially expressed genes (DEGs) for treatments with EPCD, 7kCHOL, or CHOL (all vs. VC) were selected from the total set of expressed genes using the following stringency criteria: the absolute value of “fold change” (FC) was ≥1.5, with FC defined by ±2^[log_e_ ratio], where [log_e_ ratio] = relative change in expression in natural log units, with positive or negative signs of FC matching those of the natural log (log_e_) values; adjusted *p*-values (AdjP) [25] were ≤0.0010 (four significant decimal digits). Initial gene lists compiled using these criteria were further condensed by (1) eliminating those entries lacking gene symbols or identified as “NA” in the *.cel files, and (2) removing duplicates. With regard to the latter, because of the nature of the Affymetrix chip design and the use of multiple probe sets, some genes were initially either represented in the files as within the criteria range to be considered DEGs more than once according to the above FC/AdjP limits, or they appeared as entries with FC both within and outside the selected criteria (the latter sometimes also had AdjP values but not FC well within the DEG criteria). The above strategy generated the final working gene sets (except those for the volcano plots, see further below) for all subsequent analyses.

**Figure 3:**
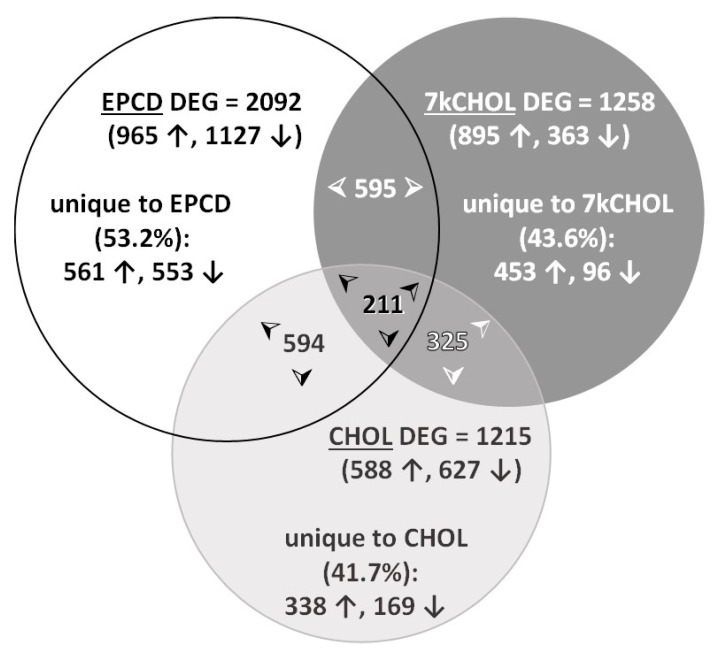
Venn diagram illustrating gene sets undergoing significant changes in regulation (|FC| ≥ 1.5, AdjP ≤ 0.0010), induced by treatments with either oxysterols (EPCD or 7kCHOL) or CHOL, vs. VC. Unique and overlapping portions of each gene set, and numbers up- or down-regulated, also are indicated.

Genes with the 20 highest positive or negative FC (these were all DEGs, by definition) are listed in Supplementary Materials (see Supplemental Table S1A,B). A complete list of all genes in the final resulting gene sets is provided in Supplementary Materials, Table S2A–C (including, for comparison, DEG results using alternative FC cutoffs of ≥ 2 and ≤ -2, for each treatment). A Venn diagram (Figure 3) illustrates the overlap of DEGs between pairs of treatment groups as well as between all three experimental conditions. Slightly more than half of the DEGs, as defined above, induced by treatment of 661W cells with EPCD were not shared with the other treatments, whereas uniquely differentially expressed genes comprised less than half of the DEG total for 7kCHOL and CHOL.

For all EPCD and CHOL DEGs, the larger total proportion were down-regulated, while the converse was the case, to a more profound degree, for 7kCHOL DEGs (Figure 3). However, for DEGs *unique* to each of the three test compounds—including 7kCHOL—the greater proportion were all *up-regulated*. Table 1 explores in greater detail the intersection of each pair of gene sets. It was obviously extremely rare to find a DEG that was up-regulated by EPCD and down-regulated by 7kCHOL. Most of the results for the subset of DEGs representing the intersection of these two oxysterol treatments were in parallel directions, which was also the case for the intersections of EPCD with CHOL, and of 7kCHOL with CHOL (Table 1). Again, the majority of DEGs shared by all three treatments displayed either up-regulation or down-regulation across the three treatment modalities (Table 1). It is also of interest that there were 20 cases where DEGs for the two oxysterols were in parallel but opposite to the FC directionality of CHOL, while 7kCHOL had opposite effects from those of EPCD and CHOL in another 15 DEGs.

#### 2.1.4. Volcano Plot

Higher stringency criteria, namely the use of weighted average difference (WAG, defined as [FC x Average Expression Level], as computed using the abovementioned software) [26], were employed to reduce the number of qualifying DEGs, thereby simplifying the visual aspects of the volcano plots. The WAG threshold was 16.0, and the AdjP cutoff was equivalent to that described above. While this method retained DEGs with the highest levels of FC, it also excluded by definition some genes with |FC| > 1.5, replacing them with some DEGs having |FC| < 1.5. Once DEGs were determined for this graphical presentation, the results were plotted in terms of FC vs. “−10 * log [AdjP]”. In general, volcano plots visually demonstrate the relationship between FC and AdjP, with higher FC generally giving rise to more statistically significant AdjP values—hence the descriptive name.

**Figure 4:**
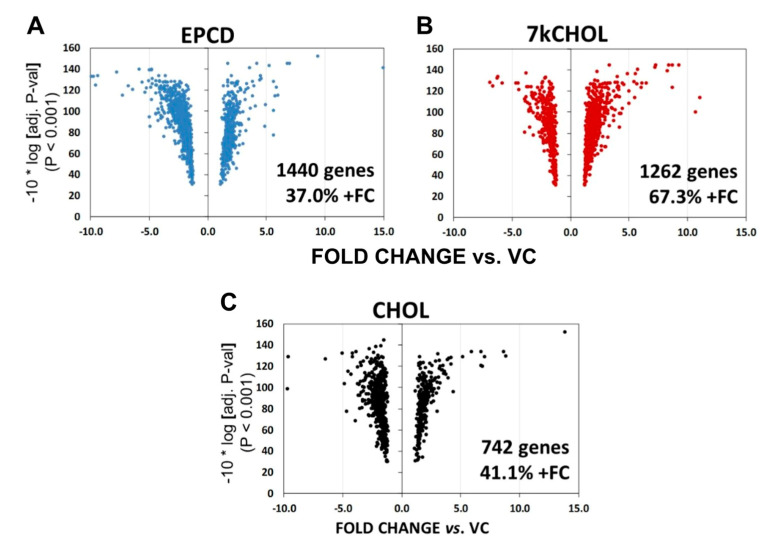
Volcano plots for the EPCD (**A**), 7kCHOL (**B**), and CHOL (**C**) treatment groups, plotted using AdjP (≤0.0010; negative logarithmic scale) as a function of FC values (for DEGs with a WAG (weighted average difference) ≥ 16.0).

As can be seen in Figure 4 (and as predicted from the Venn diagrams), all three experimental treatments generated asymmetrical volcano plots, with 7kCHOL incubations leading to an approximately 2:1 ratio of DEGs with positive vs. negative FC (Figure 4B), with the reverse trend exemplified by both EPCD and CHOL (Figure 4A,C, respectively), whose volcano plots show a majority of down-regulated genes. In this analysis, CHOL had only approximately half the number of DEGs meeting the criteria compared to the two oxysterols, and the plot for CHOL also showed less pronounced fine structure.

### 2.2. Gene Enrichment Analysis

#### 2.2.1. Rationale

Previous reports from our laboratory and those of our collaborators provided well-documented evidence for changes in protein expression, as well as for multiple modes of protein modifications, in the retinas of SLOS model rats. The dysfunctional CHOL metabolism resulting from treatment with AY9944 was shown by proteomic analysis to generate statistically significant, differential expression of proteins in functional classifications correlated with cellular homeostasis, stress, and cell death [19]. Meanwhile, a survey of the proteins from the retinal tissue of AY9944-treated animals detected a broad range of protein products with modifications emblematic of oxidative stress, in part generated by oxidized lipid byproducts [27]. Furthermore, there is ample evidence that 7kCHOL treatment of cultured cells, including RPE, affects cell signaling pathways governing responses culminating in cell survival or death, notably related to oxidative and ER stress, inflammation, and deranged mitochondrial function [28,29,30]. To further understand the molecular basis for compromised viability in photoreceptors both from SLOS model rats, and in mouse cone-derived cells (661W) treated with oxysterols as a model of SLOS [21], and, ultimately, also to obtain potential clues pertaining to the molecular pathophysiology of SLOS, we carried out gene arrays using 661W cells that were fated to lose viability as a result of exposure to our two representative 7DHC-derived oxysterols (see 4. *Methods*). We hypothesized that the set of DEGs from oxysterol- vs. VC-treated cells (also in contrast to candidate DEGs from cells exposed to CHOL, which retained viability) would be enriched in genes implicated in cell survival and homeostatic responses, in varied modes of regulated cell death, stress-activated cell signaling pathways, and, more specifically, in genes assigned to ER stress, oxidative stress, DNA damage and repair, autophagy, and organellar (e.g., mitochondrial) dysfunction.

For much of our enrichment analyses we relied on the gene ontology (GO) (and in some cases KEGG Pathway) sets utilized by the online program DAVID [31,32]. In other instances, sometimes in parallel, we carried out our own “custom” curation of signature and other relevant genes for the process or pathway of interest, in large part based on reports from the published literature that justified inclusion of these genes, including genes coding for proteins demonstrated to be involved in the processes or pathways in question. We occasionally drilled deeper into these latter gene sets by selecting genes based on information originating in published studies, characterizing or defining proteins known to *modulate* the expression, activity, or differential function of a relevant gene (or its corresponding translation product) that may not *itself* have been differentially expressed in our array data. Our findings are depicted in two kinds of charts:

(1) From the enrichment analysis were derived the vertical bar charts indicating the statistical significance (with the number of DEGs from our array also indicated) for the relevant GO terms. These charts always included separate results for DEGs with both positive and negative FC, as subgroups. In many cases, we queried both positive and negative *regulation* of appropriate pathways and processes; therefore, with the division of DEGs into positive and negative FC overlaid, the expanded results depicted were more informative and mechanistically detailed, and possibly even predictive.
(2) From our own curation, we created charts with horizontal flags representing the magnitude of expression changes for individual, selected, signature genes for the pathways/processes of interest. Genes with differential expression meeting our FC and AdjP criteria were directionally indicated by orange and blue flags to denote positive or negative FC, respectively. In addition to the ability to assess such results on a gene-by-gene basis, and to distinguish between the often contrasting pattern for the two oxysterols plus CHOL, we found that the general appearance of these charts would provide a qualitative overview of the extent of gene expression changes governing said pathway or process. Such an overall visual result is in keeping with the concept embodied in gene enrichment analysis, namely that the greater the fraction of relevant DEGs within a selected (functional, etc.) gene set is calculated to be (i.e., is overrepresented), compared to the proportion of total DEGs out of all genes in the mouse array, the more likely it is that the process/pathway in question has been affected by the experimental treatment in statistically significant fashion, and the more reliable the conclusion that it may underlie the phenotype or pathophysiology that the treatment is modeling.

#### 2.2.2. Endoplasmic Reticulum (ER) Stress

**Figure 5:**
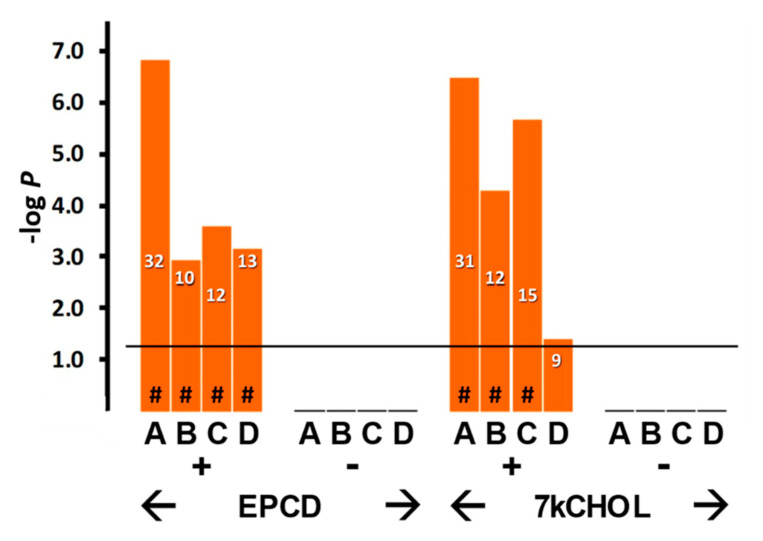
Gene enrichment analysis using the following GO terms: (**A**), response to ER stress; (**B**), intrinsic apoptotic pathway in response to ER stress; (**C**), cellular response to unfolded protein; (**D**), ERAD pathway. Solid horizontal black line in all gene enrichment charts demarcates a *p*-value (Fisher’s Exact Test) cutoff of 0.05 for statistically significant correlation for the indicated category for each DEG set. Unless otherwise indicated, all gene enrichment charts show the y axis as -log10 [*p*-value]; in this chart, note the double logarithmic scale of the y axis. Additionally, all charts show results for the two oxysterols and CHOL grouped by positive and negative FC (+ and -, respectively). In all gene enrichment charts, heavy black lines along the x axis (or absence of lettered columns) indicate that no DEGs were found for this GO term within this sample set. In this and all gene enrichment charts, on each column, numbers of DEGs identified for each GO term are shown, and “#” at the bottom of a column indicates DEG sets with enrichment scores ≥ 2.00. See main text for further interpretation.

As with most stress responses, endoplasmic reticulum stress (ER stress) may initially support a pro-survival function, but sustained activation of ER stress is generally recognized as a forerunner of cell death in all cells, including photoreceptors and other retinal neurons [33,34]. Enrichment analysis highlighted several terms associated with ER stress (Figure 5, columns A–C), but only for oxysterol treatment sets with DEGs having positive FC; those with negative FC, as well as all queries involving CHOL treatment, did not register any results in this regard. Both oxysterol-treated samples displayed strong statistical correlations for all of the categories shown except for endoplasmic reticulum-associated protein degradation (ERAD) pathway (Figure 5, Columns D; see also further below), which may indicate some mechanistic differences between EPCD and 7kCHOL in the implementation of the ubiquitin-proteasome system in the context of mitigating ER stress [35].

**Figure 6:**
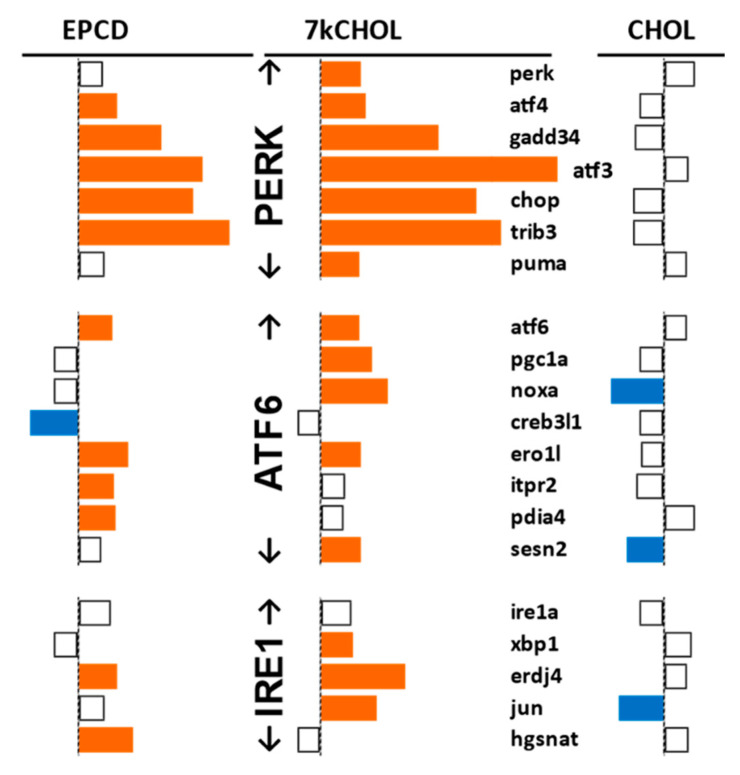
Array results for specific genes associated with the three “arms” of the canonical ER stress pathway. The horizontal extent for each flag indicates the relative positive or negative magnitude (to the right or left of center dashed vertical line, respectively) of the FC for each gene in each of the three treatment groups. White flags with black outlines denote that the FC (vs. VC) for those genes in the indicated treatment group were below our threshold criteria for a DEG. Orange and blue flags indicate DEGs meeting our criteria for both AdjP and also for FC thresholds of ≥1.5 and ≤−1.5, respectively. In charts such as those illustrated here, when results for a specific gene were obtained from more than one probe set, the FC value corresponding to the most significant AdjP value was selected, unless otherwise indicated (§, “different *p*”). Note that (with a few exceptions in charts of this kind) the omission of a nominally relevant gene within the stated category indicates that it was not differentially expressed in any of the treatment groups. Contrary to convention for mouse genes (as followed when applicable in the main text), gene symbols are provided in all lower-case lettering in this and similar charts to follow, in part for ease of design and visualization, and also because interrelationships between the genes and the proteins they represent are at times discussed in the main text in a species non-specific manner.

**Figure 7:**
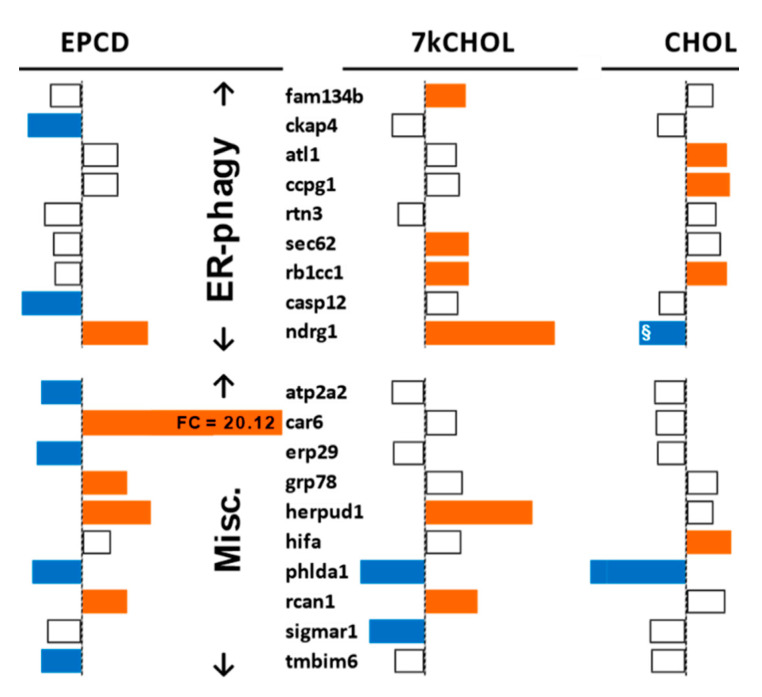
Array results for specific genes associated with autophagy of endoplasmic reticulum (“ER-phagy”), and for genes not specifically affiliated with one individual arm of the ER stress pathways illustrated in Figure 6. §, different *p* ≤ 0.001.

Further insight into the ER stress response of oxysterol-treated 661W cells was gained from the array data by curating individual genes associated with the three canonical pathways of the unfolded protein response (UPR) [36], plus non-canonical processes affiliated with ER stress (Figure 6 and Figure 7). We found DEGs in the Perk, ATF6, and Ire1 arms of the ER stress pathway; the best matches between the two oxysterols, including an extremely high level of statistical significance, were for genes in the Perk arm (Figure 6). It was noteworthy that *Perk* itself only exceeded the FC threshold in 7kCHOL-treated samples.

Both EPCD and 7kCHOL incubations up-regulated genes for proteins that drive the ATF6 and Ire1 arms, with only two of the genes of interest showing differential expression for both oxysterols. Importantly, on the basis of our threshold criteria, while *ATF6* showed positive FC in samples treated with either oxysterol, expression of *IRE1a* itself was not affected, and only 7kCHOL induced a change for *Xbp1* (Figure 6). For this profile of canonical ER stress genes, incubation of the cells with CHOL resulted in a “quiet” profile, since there were only three differentially expressed transcripts, all of which displayed down-regulation, a response opposite to the corresponding oxysterol treatments (Figure 6). Sestrin 2 (SESN2), whose gene expression was increased in 7kCHOL-treated samples but down-regulated by CHOL incubation, has been shown to be induced in the Atf6 arm of ER stress signaling [37], and to block the cytotoxic effects of sustained ER stress activation; in fact, *Sesn2* is considered a cytoprotective gene that is generally up-regulated in response to a variety of stresses, including those from oxidative challenge and DNA damage, as well as being p53-responsive [38]. In that context, it is interesting that CHOL incubation led to decreased expression of this gene (Figure 6).

The specialized category of “ER-phagy” [39] includes an additional set of genes, of which some were differentially regulated in our array analysis (Figure 7), with clear differences between all three treatment groups, especially for three genes central to this process, *Fam134b*, *Sec62*, and *Ccpg1* [40], perhaps an indication of the independent mechanistic roles played by the expressed proteins. Additionally, there was a significant oxysterol-induced up-regulation of *Ndrg1*, whose coordinately increased expression in parallel with *Trib3*, *Jun*, and *Chop* has been reported previously [41]. Otherwise, the less pronounced effect of the experimental treatments on differential expression of ER-phagy genes suggests that this process is not linked to ER stress in our experimental system.

In considering miscellaneous genes not affiliated with one specific arm of the ER stress pathway that nevertheless influence its outcome (“Misc.” group in Figure 7), it was noted that *Grp78*, one of whose several critical functions as an expressed protein is to recognize newly synthesized “client” proteins to initiate the UPR [42], was indeed differentially expressed only as a consequence of EPCD treatment (Figure 7). *Tmbim6* has been characterized as a pro-survival gene in response to ER stress [43], and here its down-regulation induced by EPCD exposure could be interpreted as emblematic of a specific avenue of cell death in our cell culture model. *Erp29* is an ER stress-responsive gene whose corresponding protein is considered neuroprotective; it has been shown to be down-regulated in the retina in human subjects with age-related macular degeneration [44]. The expression of *Car6*, the most highly up-regulated gene induced by EPCD (and by any of the three agents tested; see Supplemental Materials, Table S1A), has been shown to occur in neurons under stress conditions as a splice variant whose translation product, carbonic anhydrase (CA) VI, is retained within the cell rather than secreted, and transcribed via a CHOP/CEBPβ heterodimer (see Supplemental Materials Figure S5) that binds to an ER stress-inducible promoter; under these conditions, CA-VI is associated with cytoprotection [45,46]. 7kCHOL treatment robustly decreased expression of *Sigmar1*, whose corresponding protein has neuroprotective properties that have been documented in animal models of retinal degenerations [47]; *Sigmar1* knockdown increases CHOP expression and exacerbates ER stress [48].

**Figure 8:**
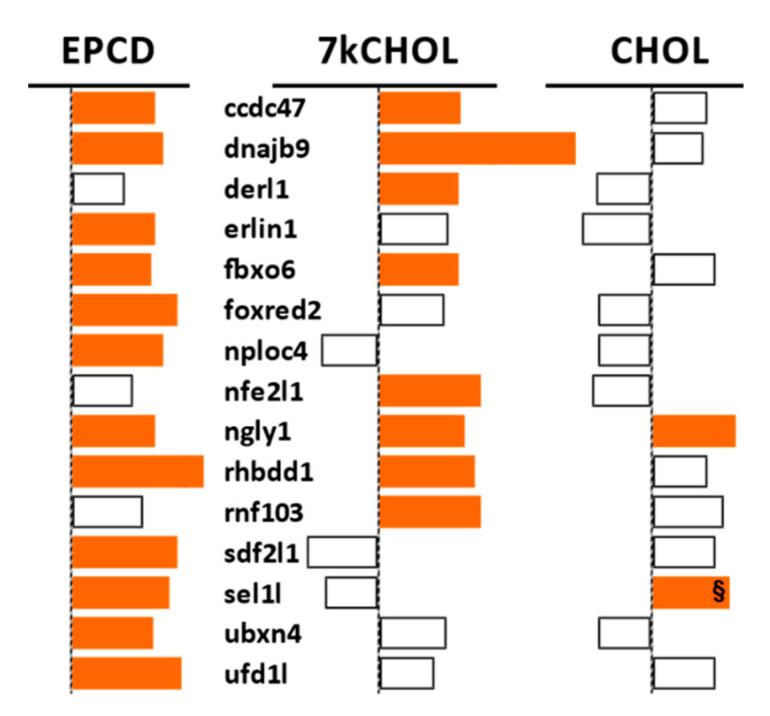
Array results for specific genes associated with the ERAD pathway. §, different *p* ≤ 0.001.

The GO:0036503 term (for ERAD pathway) corresponds to a set of genes, from which the pattern of DEGs from our three treatments (Figure 8) showed a good correlation with gene enrichment analysis for this process, depicted in Figure 5, Columns D (above), in that there were more up-regulated DEGs linked to ERAD that emerged from EPCD treatment than there were for 7kCHOL. Six of the DEGs were expressed in EPCD-incubated 661W cells only, while two were found only for 7kCHOL (Figure 8). *Ngly1*, whose corresponding cytoplasmic protein is an N-glycanase for unfolded proteins retrotranslocated from the ER [49], was up-regulated by all three compounds tested.

#### 2.2.3. Oxidative Stress

**Figure 9:**
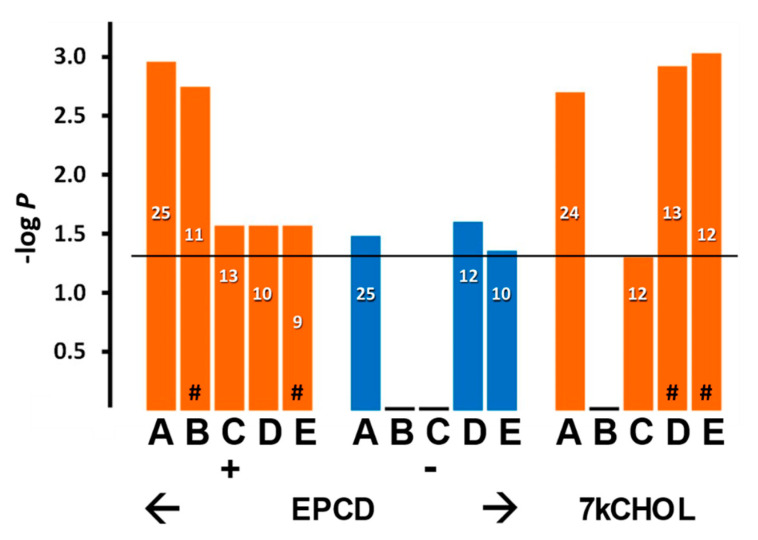
Gene enrichment analysis using the following GO terms: (**A**), cellular response to oxidative stress; (**B**), regulation of cellular response to oxidative stress; (**C**), cellular response to reactive oxygen species; (**D**), reactive oxygen species biosynthetic process; (**E**), Regulation of reactive oxygen species biosynthetic process.

Oxidative and nitrosative stress can cause biochemical modifications in the retina of the rat SLOS model [27,50], and oxidative stress has been reported in human tissue containing, and in cultured cells incubated with, oxysterols such as 7kCHOL [51,52]. Oxidative stress is linked with many of the processes embodied in the enrichment categories also interrogated for this analysis, i.e., mitochondrial dysfunction, ER stress, and DNA damage [53,54,55]. Figure 9 and Supplemental Figure S2 show the results for several GO terms within this category. Both oxysterols employed in this study generated expression of genes involved in the overall category “Cellular response to oxidative stress,” with EPCD inducing both up-regulated (+) and down-regulated (-) DEGs for this GO term (Figure 9, Columns A). Up-regulated DEGs involved in the GO term “Regulation of cellular response to oxidative stress” were recorded exclusively from EPCD-treated cells (Figure 9B). Both oxysterols induced DEGs with positive FC for “Cellular response to *reactive oxygen species”* (Figure 9C), although with not as much statistical significance as “Cellular response to *oxidative stress*,” implying that specific subtypes of redox homeostatic pathways were operating in the treated cells. 7kCHOL treatment of 661W cells led to more pronounced correlations with “Reactive oxygen species biosynthetic process” (Figure 9C,D), including positive regulation, compared to EPCD, here suggesting, according to the full definition of this GO term, that 7kCHOL was more prone to generate reactive oxygen species, in contrast to other types of redox dysregulation. Only EPCD treatment induced DEGs with negative FC for any of the terms illustrated in Figure 9, although the *p*-values were not as significant as for positively regulated genes (negative results for 7kCHOL not shown; Figure 9). Samples incubated with CHOL were not enriched when queried with any of the GO terms in Figure 9, indicating that this compound did not elevate reactive oxygen species nor induce oxidative stress.

Nitrosative stress enrichment results are presented in Supplemental Materials, Secton S.2.2.3., and shown in Figure S2. Results for compilation of genes for antioxidant enzymes, including Nrf2 targets, and other regulators, are presented in Supplemental Materials, Secton S.2.2.3.1, and Figure S3.

#### 2.2.4. Autophagy

**Figure 10:**
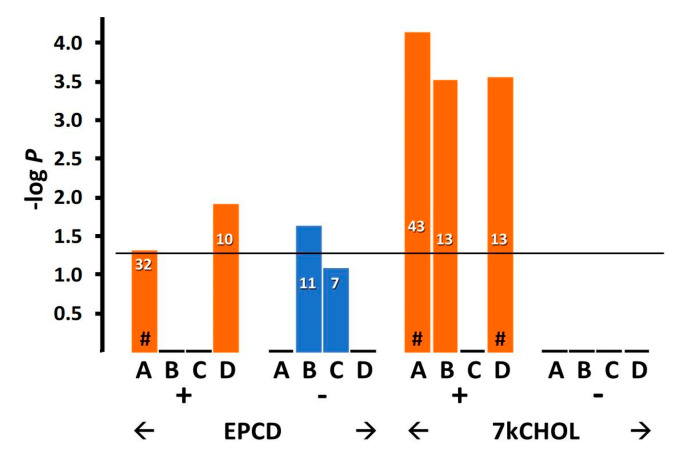
Gene enrichment analysis using the following GO terms: (**A**), autophagy; (**B**), positive regulation of autophagy; (**C**), negative regulation of autophagy; (**D**), autophagosome.

**Figure 11:**
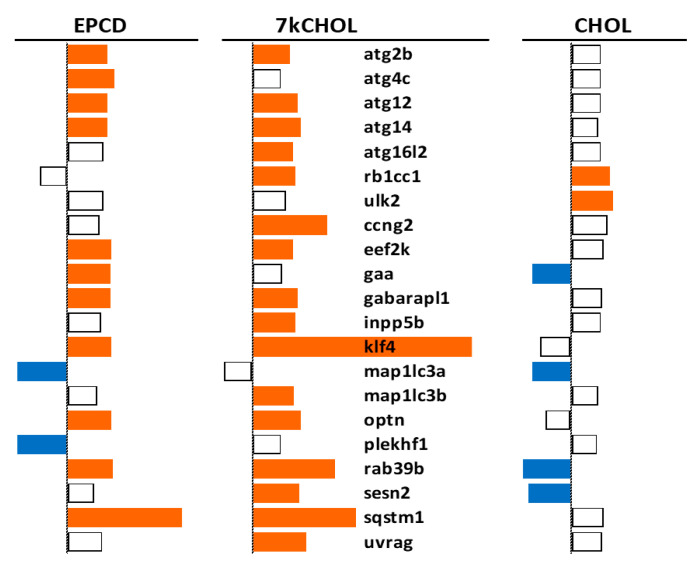
Array results for specific genes associated with autophagy.

**Figure 12:**
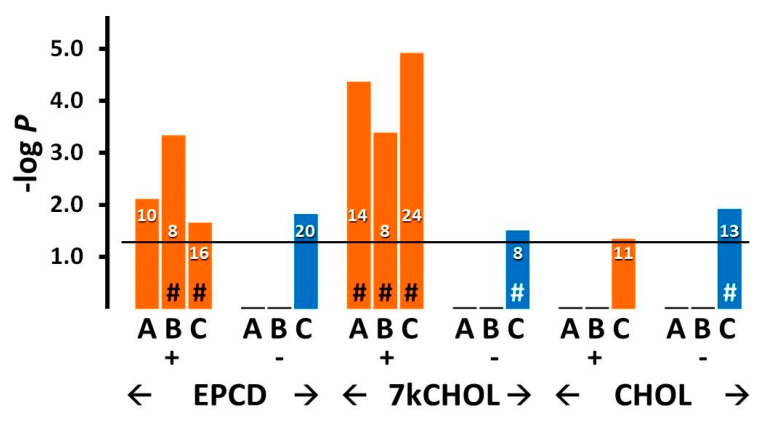
Gene enrichment analysis using the following GO terms: (**A**), TOR signaling; (**B**), negative regulation of TOR signaling; (**C**), cellular response to insulin stimulation.

**Figure 13:**
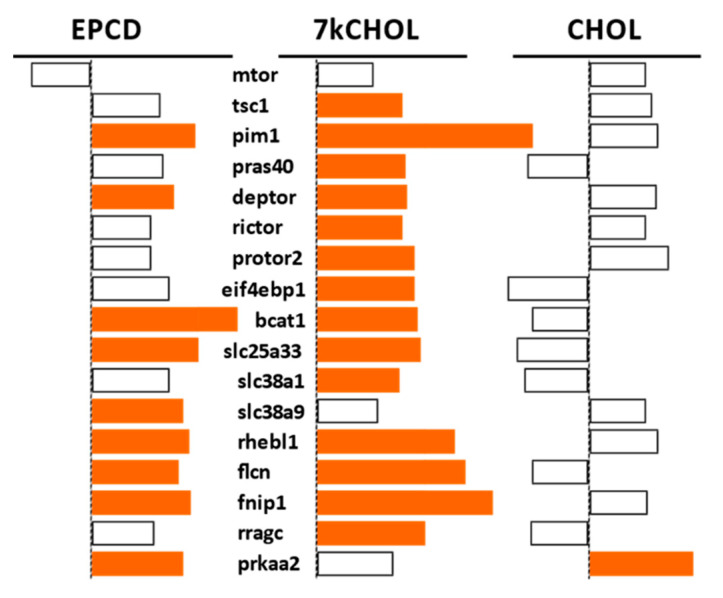
Array results for specific genes associated with mTorC 1/2 signaling and regulation.

Autophagy is a key stress response in neurons, and enhanced autophagy has been proposed to be a protective adaptation in several neurodegenerative diseases [56,57]. While its direct role in apoptosis is disputed, there is agreement that autophagy often accompanies cell death [58]. In the retina, autophagic mechanisms contribute to both homeostatic functions and also to the pathophysiology of retinal degenerations [59]. Our laboratory previously described dysregulation of components of the autophagosomal pathway in the RPE of the rat SLOS model [60]. Additionally, using cultured induced pluripotent stem cell-derived human RPE bearing a SLOS mutation, impaired phagosome maturation was described that reflected a failure of primary phagosomes to fuse with lysosomes [60]. An interest in enrichment of genes from our arrays, whose differential expression could influence autophagy in the 661W cells under treatment conditions employed by us (Figure 10 and Figure 11), was predicted by the knowledge of cross-talk between cellular stress responses, and further stimulated by the results for mTorc1-associated genes (Figure 12 and Figure 13, below), since mTorc1 expression and activity has been shown to negatively regulate autophagy [61].

Somewhat contrasting overall patterns of gene expression elicited by the two oxysterols were indicated by enrichment analysis using GO terms related to autophagy (Figure 10). For the broad heading of “Autophagy” (Figure 10, Column A), a more significant *p*-value, and more up-regulated DEGs were elicited by incubation with 7kCHOL, compared to EPCD; also, DEGs with positive FC for only this oxysterol yielded significant results for positive regulation of autophagy (Figure 10B). On the other hand, while with 7kCHOL treatment no negatively-regulated genes registered for any gene enrichment category shown in Figure 10, down- regulated DEGs with a statistically significant impact with respect to positive regulation of autophagy were only induced by EPCD (Figure 10B). There were no DEGs, displaying positive or negative FC, from either of the oxysterol-treated samples that showed a significant correlation with *negative* regulation of autophagy (Figure 10C). Importantly, no enrichment terms related to autophagy could be found for the set of DEGs from CHOL-treated cells (not shown). Both oxysterol treatments—with a more impressive *p*-value and more total DEGs for 7kCHOL treatment—were positively correlated with the subcategory of “Autophagosome,” as distinct from the more general enrichment term of “Autophagy” (Figure 10, Columns D vs. A).

Results for representative autophagy genes from 661W cells exposed to oxysterols agreed with the enrichment analysis, suggesting overall increased engagement of the autophagosomal system (Figure 11). Oxysterols profoundly up-regulated *Sqstm1*, also known as its protein product, p62, which (like OPTN [62]) recognizes ubiquitinylated cargo to be linked to nascent autophagophores [63,64]. Numerous genes identified as being from the “Autophagy Related” (Atg) functional class, whose protein products are involved with formation of the phagophore as an initial autophagic event [65], were also up-regulated by EPCD and/or 7kCHOL (Figure 11). Among the positive effectors of the autophagic process is GABARAPL1, the most highly expressed member of the Atg8 protein family in the CNS [66], whose gene was up-regulated by both oxysterols. For other Atg8 family members, in contrast, there was a distinction between the two oxysterols; *Map1lc3a* was down-regulated by EPCD, and only 7kCHOL treatment differentially regulated *Map1lc3b* (Figure 11). Expression of *Klf4*, whose corresponding protein function appears to be critical for autophagy as a pro-survival response to DNA damage [67], was increased by oxysterols, and with especially high magnitude by 7kCHOL; this gene exhibited the fourth highest positive FC induced by the latter oxysterol (Supplementary Materials, Table S1A). While most of the DEGs illustrated in Figure 11 whose translation products associated with autophagy have been documented to participate in the earlier steps in this process, UVRAG, one of whose roles is to regulate fusion of the autophagophore with lysosomes [68], was also transcriptionally up-regulated by 7kCHOL but not EPCD (Figure 11). ULK1 and ULK2 are components of the Atg1 complex, subserving multiple regulatory roles in the early formation of autophagosomes, including feedback interactions with mTorc1 and AMPK [69,70]. Neither *Ulk1* nor *Ulk2* were affected by oxysterols, but *Ulk2* was one of only two autophagy genes that were up-regulated by CHOL (Figure 11). Also of interest was the DEG *Eef2k*, whose product is a transducer of ER stress-induced autophagy, the activity of which undergoes positive modulation by Ddit4 up-regulation, via inhibition of mTorc1, and also by the PRKAA2 subunit of AMPK [71]. As an example supporting the contrasting enrichment results between the treatment groups for autophagy (Figure 10), the transcript representing SESN2, whose demonstrated inhibition of mTorc1 results in positive regulation of autophagy [72], was not a DEG in EPCD-treated samples, but was differentially expressed in opposite manner by 7kCHOL and CHOL (Figure 11).

Array results for genes affecting the macroautophagic process of mitophagy are presented in Supplemental Materials Section S.2.2.4. (Including Figure S4).

#### 2.2.5. mTORC Pathways

Because the mTORC 1/2 signaling pathways have been shown to impact cell survival, including playing a role in neurodegeneration [73,74,75], we next evaluated enrichment terms relevant for these in our gene array results (Figure 12). The set of up-regulated, but not down-regulated, oxysterol-induced DEGs exhibited a statistically significant correlation for the GO term “Tor signaling,” which includes genes for both mTorc1 and mTorc2 (Figure 12A). At a more specific level, “Negative regulation of Tor signaling” enrichment was found in oxysterol-treated samples, and only for up-regulated DEGs (Figure 12B); no DEGs registered when interrogated for *positive* regulation of this pathway (not shown). Receptor-mediated activation of mTorc1 by insulin (and insulin-like growth factors) can occur via the PI3K/Akt signaling pathway [76], and while all three treatment schedules yielded DEGs with both positive and negative impact on the response to insulin stimulation, the balance for 7kCHOL incubation was towards more up-regulated DEGs, with a lower *p*-value, in this category (Figure 12C).

Individual DEGs for components of mTor complexes 1 or 2, as well as for upstream regulators and downstream effectors—of mTorc1 in particular—are illustrated in Figure 13. There were no significant expression changes for the *Mtor* gene itself in any of the three treatment groups. However, it is easily discernible that treatment of 661W cells with EPCD or 7kCHOL, in contrast to CHOL, caused an overall pattern of up-regulation of gene expression of the listed genes, that would be expected to affect the integrity of mTorc operation and signaling within the cell. It is difficult and risky to interpret the transcriptional effects of oxysterols on net activity of mTorc1 as up- or down-regulated solely based on the results shown in Figure 13, because of the complexity of all of the interactions involved, namely both positive and negative regulation at the protein level, including the negative feedback of mTorc1 on Akt [77].

It is intriguing in this respect that 7kCHOL up-regulated *Rictor* and *Protor2*, both specific for mTorc2, generally considered to be an activating factor for Akt, and therefore whose potentiation would be assumed to attenuate cell death processes [78]. Engagement and interactions of additional mTORC pathway DEGs depicted in Figure 13 have been described [79,80,81,82,83,84,85,86,87,88].

#### 2.2.6. DNA Damage and Repair

**Figure 14:**
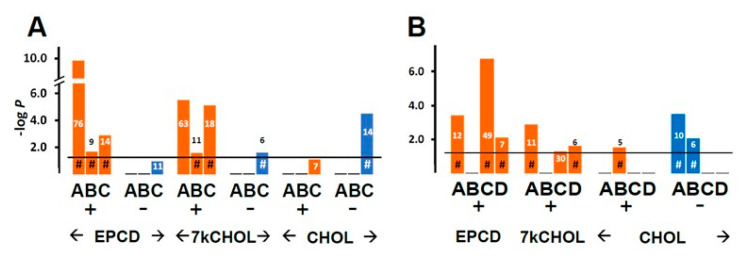
(**A**), Gene enrichment analysis using the following GO terms: (**A**), cellular response to DNA damage stimulus; (**B**), positive regulation of response to DNA damage stimulus; (**C**), intrinsic apoptotic signaling pathway in response to DNA damage. (**B**), gene enrichment analysis using the following GO terms: (**A**), signal transduction in response to DNA damage; (**B**), negative regulation of response to DNA damage stimulus; (**C**), DNA repair; (**D**), positive regulation of DNA repair.

**Figure 15:**
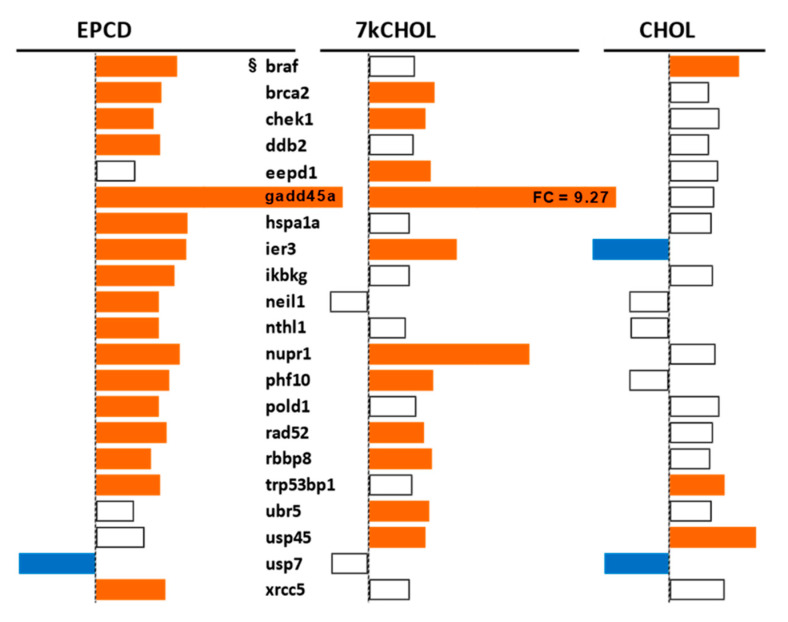
Array results for specific genes associated with DNA damage and repair.

Results for enrichment analysis using GO terms related to DNA damage and repair are presented in Figure 14A,B. DEGs with positive FC (“+”) assigned in the oxysterol treatment groups manifested a highly statistically significant correlation for the term “Cellular response to DNA damage,” with 76 and 63 genes compiled in this category for EPCD and 7kCHOL, respectively (Figure 14A, Columns A). Further analysis of DEGs having positive FC induced by oxysterol treatments indicated, first, significant *p*-values for *positive* regulation of the DNA damage response, as opposed to no results for *negative* regulation of the DNA damage response (Figure 14A, Columns B vs. Figure 14B, Columns B), and second, a role for DNA damage in the cell death of the oxysterol-treated 661W cells (Figure 14A, Columns C). By comparison, only the set of down-regulated genes differentially expressed by CHOL-treated cells registered a significant *p*-value for GO term “Intrinsic apoptotic signaling in response to DNA damage” (Figure 14A, Columns C), which was the opposite of the apparent pattern for the two oxysterols, and likely correlated with the results in Figure 15 and the obvious sustained viability of the cells incubated with CHOL. In like manner, while correlation with DNA damage-generated signal transduction cascade genes was only observed with oxysterol-induced—but not CHOL-treated—up-regulated DEGs, CHOL treatment did result in a significant correlation for down-regulated DEGs with this term (Figure 14B, Columns A). Under the heading of DNA repair, the correlation for up-regulated genes was much more pronounced for EPCD treatment vs. 7kCHOL (Figure 14B, Columns C), while both oxysterols showed significant *p*-values for the subcategory of positive regulation of DNA repair (Figure 14B, Columns D). In the context of results taken together for this general enrichment category, and considering that CHOL treatment did not engender cell death, it was not surprising that samples for CHOL treatment did not yield any enrichment results for DNA repair (Figure 14B, Columns C, D).

Figure 15 illustrates the effects of the experimental treatments on representative DEGs that may be activated, and coding for proteins that are active, in the cellular responses to DNA damage, including those involved in the signaling underlying this response, or those promoting repair of damaged DNA. Among the functions for gene products included in this list include sensing of unstable base pairs, removal of histones to expedite repair of DNA, nucleotide/base excision, and double-strand break repair [89,90,91]. Some of these genes (e.g., *Usp7*, *Ddb2*) have ubiquitination or de-ubiquitinylation activities that are integral to their DNA damage recognition and repair functions aside from their more canonical relation to the proteasomal degradation pathway [92,93]. Both of the oxysterol treatments, but not incubation with CHOL, induced prominent up-regulation of *Gadd45a*, coding for an important sensor of DNA damage and other forms of cellular stress with downstream pro-survival effects [94], along with *Ier3* [95], which was in fact down-regulated in CHOL-treated samples.

### 2.3. Increased Expression of Proteins Coded by Selected DEGs Following Oxysterol Treatments

#### 2.3.1. Introduction

Transcriptional changes of individual genes—specifically for the DEGs reported here, with |FC| ≥ 1.5—may only be functionally significant if they reflect detectable positive modulations in corresponding protein abundance and/or activity. We used confocal immunofluorescence microscopy of 661W cells treated with either EPCD or 7kCHOL to evaluate, for selected genes that are critical to and representative of stress response pathways, the match between differential expression results in the gene array and for immunoreactivity that might reflect parallel changes in translation driven by modulation of transcription (and/or of other events affecting mRNA abundance). Comparisons were made for each oxysterol treatment with the appropriate vehicle control. In some samples, the concentrations of the oxysterols were different from what was employed for the array study, but as with the latter, the time frame for fixation and processing of the cultures for immunofluorescence was observed to maintain the morphological integrity of the majority of cells before extensive loss of viability—and immunoreactivity—had occurred. Effects of CHOL exposure were not assessed in this set of experiments.

**Figure 16:**
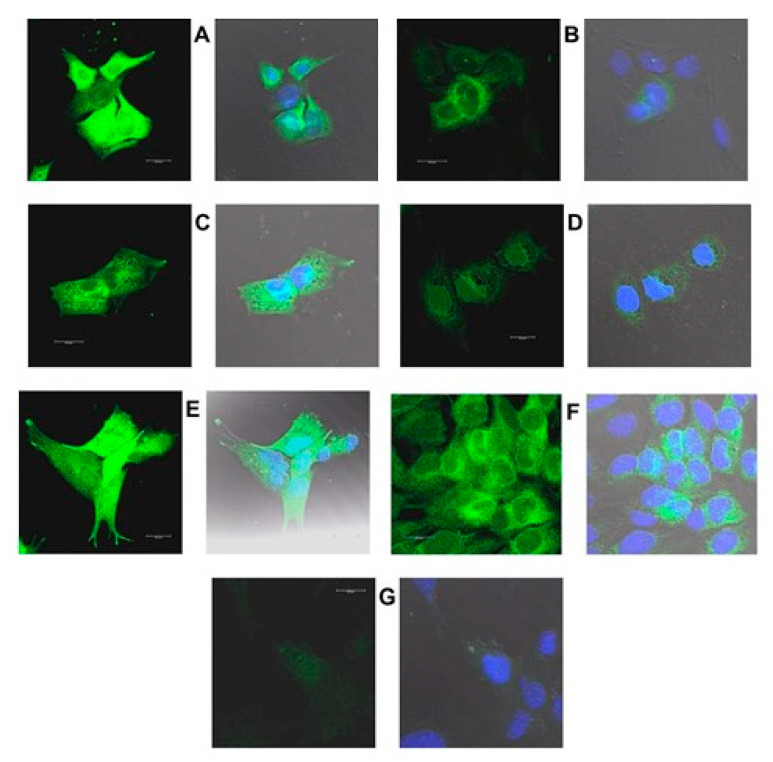
(**A**–**G**): Immunoreactivity for heme oxygenase-1 (HMOX1). (**A**–**D**), 661W cells were fixed with methacarn; (**E**–**G**), cells fixed with formaldehyde. (**A**): Cells treated with 6 μM EPCD. Intense fluorescent signal indicates HMOX1 immunoreactivity present in cytoplasm and nuclei in 4 of 5 cells in the microscopic field of view. (**B**): Corresponding treatment with DMSO resulted in comparatively much lower, predominantly cytoplasmic immunoreactivity for HMOX1. (**C**): 20 μM 7kCHOL treatment. Cytoplasm exhibits vesicular pattern of HMOX1 immunoreactivity, and also signal in nuclei, indicated by partial overlap of green pseudocolor with blue DAPI fluorescence. (**D**): hpβCD VC sample shows very low intensity cytoplasmic immunoreactivity for HMOX1, with no signal in nuclei. (**E**): In this field of cells treated with 20 μM 7kCHOL, a range of immunofluorescent intensities was recorded, with extensive co-localization of nuclear signal (blue-green pseudocolor) for both HMOX1 with DAPI. (**F**): hpβCD VC treatment resulted in baseline HMOX1 immunoreactivity restricted to a perinuclear, vesicular pattern, with minimal nuclear localization. (**G**): Absence of specific immunoreactivity in 7kCHOL-treated 661W culture processed with normal rabbit IgG as primary antibody negative control.

**Figure 17:**
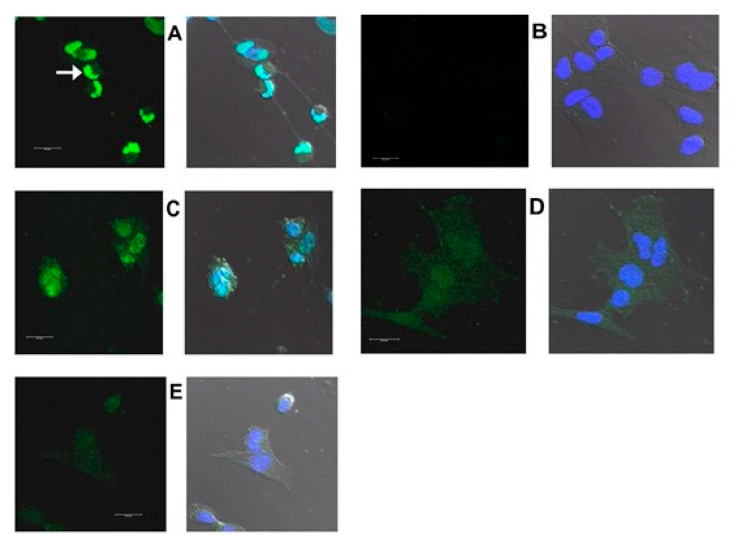
(**A**–**D**): Immunoreactivity for CHOP. Formaldehyde fixation was employed for all samples in this figure. (**A**): Cells treated with 8 μM EPCD. Intense CHOP immunoreactive signal restricted to contracted, comma-shaped nuclei (left, arrow), overlapping with DAPI staining (right) to generate blue-green pseudocolor. (**B**): For DMSO vehicle control treatments, no specific signal was observed, with only nuclear DAPI fluorescence. (**C**): Treatment with 25 μM 7kCHOL resulted in primarily nuclear CHOP immunolocalization. (**D**): Cells treated with hpβCD VC reveal only non-specific background fluorescence, and only DAPI fluorescence (blue pseudocolor) in nuclei. (**E**): Normal mouse IgG substituted as reagent negative control in place of specific antibody against CHOP, showing non-specific background fluorescence comparable to that in (**D**).

**Figure 18:**
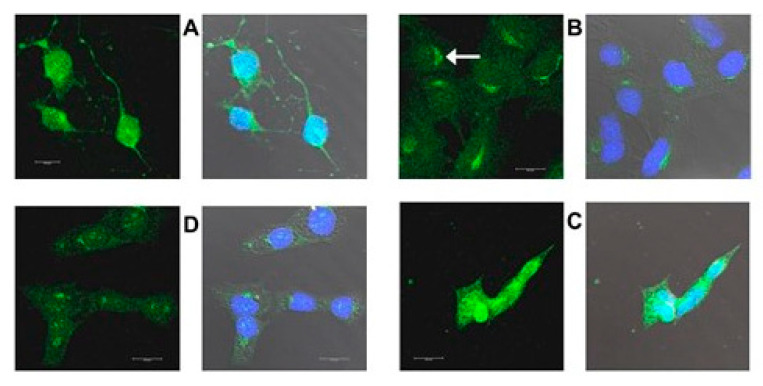
(**A**–**D**): Immunoreactivity for TRIB3. All 661W cells presented in this figure were fixed with methacarn. (**A**): In 10 μM EPCD-treated cells, immunoreactivity for TRIB3 was detected in neuritic extensions as well as in somata, with occasional nuclear signal (overlap with DAPI stain). (**B**): DMSO control in place of EPCD treatment. TRIB3 signal limited to perinuclear region (arrow), in cells with a more “spread” morphology compared to (**A**). Nuclei exhibit only DAPI immunofluorescence (dark blue pseudocolor). (**C**): 10 μM 7kCHOL incubation. Amorphous TRIB3 detected in cytoplasm and also nuclei. (**D**): hpβCD VC treatment resulted in only sparse, punctate TRIB3 immunoreaction found perinuclearly and with occasional colocalization with DAPI nuclear stain (right).

**Figure 19:**
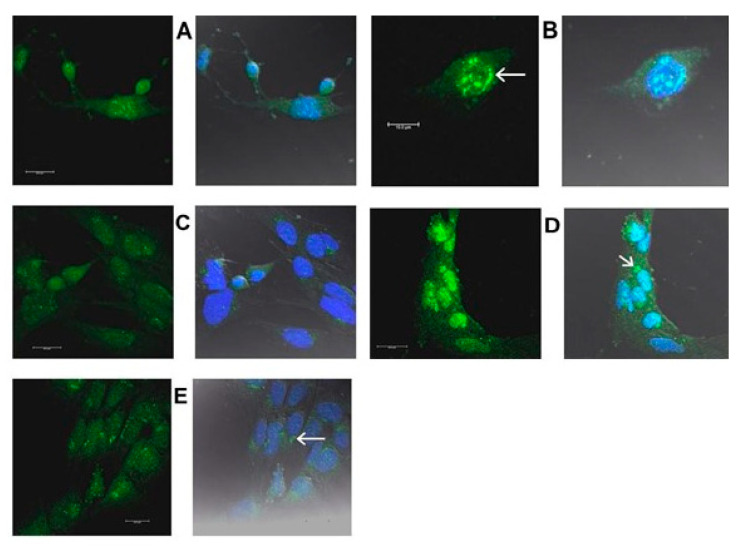
(**A**–**E**): Immunolocalization of HERPUD1. (**A**–**C**), 661W cells were fixed with methacarn; (**D**,**E**), cells fixed with formaldehyde. (**A**,**B**): For 8 μM EPCD-treated 661W cells, there were large, dense aggregates of HERPUD1 immunoreactivity (green pseudocolor in left images, and blue-green superimposition with DAPI fluorescence) detected in the nuclear zones (arrow in **B**). Bar = 10 μm in (**B**). (**C**): DMSO (VC) substituted for EPCD incubation. Predominantly non-specific background fluorescence, with only sparse, punctate immunoreaction in the vicinity of nuclei. Nuclei exhibit only DAPI staining (blue pseudocolor). (**D**): 661W cells treated with 25 μM 7kCHOL display both nuclear and juxtanuclear (arrow) HERPUD1 immunofluorescent signal, the former as aggregates of various sizes, coinciding partly with DAPI fluorescence, along with less intense, punctate cytoplasmic immunoreactivity. (**E**): Cells incubated with hpβCD VC demonstrate a focus of HERPUD1-specific immunofluorescence above background near nuclei (arrow), with some punctate cytoplasmic immunoreactivity; nuclei show primarily DAPI staining only, compared to the 7kCHOL-treated sample (**D**).

Figure 16, Figure 17, Figure 18 and Figure 19 are confocal immunofluorescence micrographs of 661W cells cultured on Chamberslides and treated with either: EPCD; DMSO, the vehicle control (VC) for EPCD; 7kCHOL; or hydroxypropyl-β-cyclodextrin (hpβCD), the corresponding VC for 7kCHOL. Results are presented as paired images, with left-hand panels showing specific immunofluorescence only, and right-hand panels adding DAPI (nuclear) fluorescence, with differential interference contrast (DIC) image fields superimposed. [Unless otherwise indicated, all magnification scale bars are 20 μm]. 

#### 2.3.2. HMOX1

661W cells incubated overnight (less than 24 h) with 6 μM EPCD displayed intense immunoreactivity for HMOX1 (heme oxygenase-1) (Figure 16A), compared with DMSO (VC) alone (Figure 16B). (In each pair of images, the result for detection of AlexaFluor^®^ 488 fluorescence is on the left, and the right image is the overlay of the AF488 detection channel together with that for detection of DAPI, and with the DIC field.) For both oxysterols, as well as control treatment/processing, sample images using the λ = 488 nm laser line (left image of each pair) represent the same number of identical thickness optical sections for maximum projections, and also equivalent laser power, excitation and emission windows, gain, offset, and post-capture intensification. For HMOX1 immunoreactivity, there appeared to be some heterogeneity, as evidenced by one of the cells in the field depicted in Figure 16A. 7kCHOL treatment at 20 μM also increased the signal for HMOX1 compared to incubation with the corresponding VC (hpβCD), the intensity of signal being higher in this case after formaldehyde fixation (Figure 16C–F). Again, there was some heterogeneity of this intensity demonstrated among individual 661W cells within one observational field (Figure 16E). Some presumably constitutive expression of HMOX1 in vehicle-treated cells was apparent, since the immunofluorescence intensities were distinctly above the nominally undetectable background levels obtained when normal (non-specific) rabbit IgG was employed as primary antibody (Figure 16B,D,F,G). (Note that except for CHOP (below), Figure 16G serves as an operational control for all succeeding confocal microscopy results.) When immunofluorescence intensity reached its highest levels within cells, following either EPCD or 7kCHOL treatments (Figure 16A,E), the yellow-green pseudocolor (from the 461 nm channel) blended with the dark blue DAPI pseudocolor (using the 405 nm channel) to yield, either wholly or partially, a corresponding light blue nuclear label in the composite z-axis maximum projections (which also incorporated the DIC image) (also visible in Figure 16C); this result was largely or entirely absent in operationally equivalent images from vehicle-treated cells (Figure 16B,D,F). This overlap may reflect immunodetection of a C-terminal proteolytically cleaved form of HMOX1 that is released from the ER, and trafficked to the nucleus, where it is transcriptionally active under conditions of ER stress [96].

#### 2.3.3. CHOP

Overnight treatment with 8 μM EPCD resulted in CHOP (C/EBP homologous protein) immunoreactivity in formaldehyde-fixed 661W, that fully overlapped with nuclear DAPI labeling (Figure 17A), while the CHOP signal was absent from VC-treated cells (Figure 17B). The EPCD-elicited pattern is in keeping with the ER stress-associated transcription factor functionality of CHOP [34]. CHOP immunoreactivity also appeared to be primarily nuclear following acute treatment of 661W cells with 25 μM 7kCHOL, likewise using formaldehyde fixation (Figure 17C), compared with no signal above background in VC-treated preparations (Figure 17D). Since the anti-CHOP antibody was a monoclonal generated in mouse, normal mouse IgG was employed as a control primary antibody (Figure 17E), yielding no demonstrable specific immunofluorescence.

#### 2.3.4. TRIB3

661W cells exposed to 10 μM EPCD responded with widely distributed punctate labeling using anti-TRIB3 (tribbles homolog 3) antibody, visualized in several subcellular compartments: neurites, cell body cytoplasm, as well as nuclei (Figure 18A). This was in contrast to immunoreactivity limited to small perinuclear patches seen in VC-treated cells (Figure 18B); otherwise, there was no co-localization of TRIB with the dark blue pseudocolor of the DAPI-stained nuclei as a result of VC treatment. The specific immunofluorescence pattern observed in 7kCHOL-treated cells (both these and EPCD-treated cells had methacarn as primary fixative) was similar in terms of the punctate pattern in the cytoplasm, but was exemplified in at least one cell by homogeneous nuclear labeling that overlapped with DAPI labeling (Figure 18C), as noted for CHOP (Section 2.3.3). As observed for DMSO VC (Figure 18B), perinuclear immunolabeling was seen in the matching hpβCD-treated VC cells (Figure 18D), as well as additional specific fluorescent immunoreactive puncta that were never as dense or intense as those of oxysterol-treated cells.

#### 2.3.5. HERPUD1 

Moderately intense immunofluorescent label for HERPUD1 (homocysteine-responsive ER protein with ubiquitin-like domain 1) that ranged from small puncta to larger aggregates, was associated with cell nuclei in 661W cells incubated with 8 μM EPCD and fixed in methacarn (Figure 19A,B). EPCD-treated cells also displayed some sparse cytoplasmic immunoreactivity above background, similar to the low level of expression elicited by VC treatment (Figure 19C). In the latter case, the blue pseudocolor representing nuclear DAPI staining was not brightened by any superimposed HERPUD1 immunoreactive colocalization. Acute treatment with 7kCHOL followed by formaldehyde fixation provided a result qualitatively similar to that from EPCD treatment, except that the intensity of the signal for HERPUD1 was much greater (Figure 19D); the HERPUD1 immunoreactivity was apparent not only associated with the nuclei of the cells, but also as a focus of bright spots near the nuclei. The matching VC-treated cells also revealed this latter juxtanuclear immunofluorescence, as well as sparse, mostly perinuclear localization of HERPUD1 immunoreactivity (Figure 19E).

## 3. Discussion

Our purpose in undertaking this gene array study was, in part, to categorize gene expression in a cell culture model of a neurological disease, namely SLOS, and to advance knowledge regarding the molecular pathophysiology of SLOS. This was accomplished using an otherwise “wild-type” neuronal cell line that was exposed to purified compounds known to be formed endogenously in SLOS patients (by oxidation or metabolism of 7DHC [97,98]), one of them (EPCD) being unique to this inherited disease [99]. Our results support the hypothesis that the significant changes observed using enrichment analysis, plus documentation of differentially expressed signature genes, would provide new information regarding the etiology and disease course of SLOS, in terms of the relationship between the genotype (loss of function of *DHCR7*) and phenotype (the results of changes in the transcriptome) of this disease at the molecular level. Since our inaugural studies demonstrated that reproducing the genetic defect of SLOS by chemically inhibiting the final step of CHOL biosynthesis, using the rat SLOS model [16], also caused retinal degeneration—manifested most prominently in the outer nuclear layer—we further intended to gain insights into degeneration, cell death, and survival of photoreceptors by utilizing 661W, a mouse cone-derived photoreceptor cell line [100], for this series of investigations [21]. Our use of oxysterols derived from 7DHC to challenge the cultured cells induced a wide range of cell stresses and challenges to homeostasis that have also been documented in retinal disease and damage [35,69,101,102,103,104,105].

As with all transcriptomic analyses, various levels of validation are required to lend further predictive value and translational relevance to this study. We fulfilled this obligation at different steps of the project, including genotypic and phenotypic characterization of the in vitro model [21], experimental design (including selection of controls), integrity of samples (in part demonstrated by the PCA), processing of raw data, stringency thresholds, confirmation of underlying hypotheses (i.e., predicted canonical stress pathways emerging in oxysterol- but not CHOL-treated cells), and correlation between selected DEGs and their expression at the protein level as demonstrated by immunohistochemistry. Where stringency parameters to define DEGs is concerned, ours were not only expedient and arbitrary, as generally acknowledged [106], but also within conventionally applied boundaries [31,106,107]; notably, in any of the three gene sets corresponding to the experimental treatments, fewer than 0.6% of the genes initially surpassing the |FC| ≥ 1.5 cutoff also demonstrated AdjP of >0.0010 (Supplementary Materials, Table S3).

For reasons of economy of cost and analysis, we chose to use samples treated with hpβCD as the VC with which to compare the three (oxy)sterol treatments to generate the sets of DEGs, realizing that the most immediate comparison would be with the 7kCHOL-treated cells, for which hpβCD was indeed the vehicle. It was also acknowledged that while cyclodextrins may act as a sink for sterols supplied in solution to cultured cells, the extracellular dynamics of EPCD molecules, with DMSO as vehicle, once they were at final concentration in the IM for cell incubation, are unknown. Interestingly in this regard, we noted that 7kCHOL exerted cytotoxic efficacy with much more rapid dynamics than EPCD (Figure 1), even though EPCD is more potent. Because in practice DMSO was a more reliable vehicle for EPCD, we also used DMSO as vehicle for CHOL; in this facet of the design, we reasoned that at least at the level of individual DEGs (the genes being selected, as illustrated in our “flag charts,” in a custom curation representing pathways and processes of interest), we could demonstrate differences in gene expression between treatments that were either ultimately cytotoxic (EPCD), or were comparable in regards to growth and morphological phenotype with the hpβCD VC-treated cells (CHOL-treated samples). It should be emphasized that CHOL was selected more as a non-toxic control sterol, technically administered to the cultures in non-physiological fashion, rather than as a potential metabolite (e.g., receptor-mediated via lipoprotein). In fact, in analyses of CHOL biosynthesis pathway genes that will be reported separately, we found that there was virtually no down-regulation of these genes vs. VC, contrary to what was expected based on previously published landmark reports (e.g., [108]; data not shown).

Because of the increased specificity and rigor, qRT-PCR validation of highlighted genes that exemplify enriched pathways and processes are extremely useful when applied to transcriptomic studies. Given the scope of this investigation, it would have been an impossible task to validate every DEG of interest by any means. Under steady state conditions, it is accepted that there is a high correlation between gene transcript and protein copy numbers, independent of cell or tissue origin, and, indeed, with sufficient correlation between mouse and human CNS [109,110]. However, our acute challenge of the 661W cells with experimental compounds certainly qualifies as a “state transition,” necessitating considerations of the distinction between positive and negative gene regulatory responses, translation delays, and targeted degradation of transcripts [36,111]. Since evaluation of transcriptomic results presumes in part an interest in effects on the resulting proteome, we pursued direct correlation between some of the most highly up-regulated DEGs, with signature status for some of the prominent enrichment pathways identified, and the expression of their corresponding proteins in oxysterol-treated 661W cells, in terms of intensity and subcellular localization, assessed by means of immunohistochemistry.

Our immunofluorescence results indicate that there was some baseline expression of HMOX1 in the cultured 661W cells (as evidenced by samples treated with VC), but in parallel with the pronounced transcriptional up-regulation for *Hmox1* (Table S4), oxysterol treatments produced a notable increase in immunoreactivity for HMOX1. *Hmox1* codes for one of two decyclizing heme oxygenase enzymes (HMOX1 and HMOX2), of which the latter (not a DEG, not shown) may be constitutively expressed in neurons [112], while HMOX1 is an inducible form. In a variety of cells and tissues, *HMOX1*/HMOX1 gene and protein expression are positively modulated by a wide range of factors, and to a great extent by stressors such as those resulting from oxidative, hypoxic, inflammatory, or electrophilic stimuli, including ER stress and reduced glutathione (GSH) depletion [113,114,115]; defined agents promoting up-regulation of *Hmox1* include heavy metals and xenobiotics [116]. Oxidized low-density lipoprotein, which is known to contain 7kCHOL, has been shown to induce HMOX1 in cultured vascular smooth muscle cells [117], and up-regulation of HMOX1 by pure 7kCHOL has only been documented for this cell type [118]; this is the first instance demonstrating differential gene expression of *Hmox1* utilizing oxysterol-treated cells of neural origin. Not surprisingly, transcriptional control sites in the *Hmox1* promoter are exceptionally numerous and diverse [119], and include the antioxidant response element (ARE) with which NRF2 (the product of *Nfe2l2*) interacts [120,121]. In partial contrast to its participation in the homeostasis of erythroid cells, where it is necessary for disposal of unconjugated heme [122], up-regulated HMOX1 activity in neurons has been documented as cytoprotective, by virtue of the actions of its immediate and downstream products, carbon monoxide (CO), and bilirubin (BLRB), respectively. The gasotransmitter CO has anti-inflammatory and anti-apoptotic properties [123], and exogenous CO has been demonstrated to be neuroprotective, by virtue of activation of guanylyl cyclases and mitogen-activated protein kinases, thus far in retinal ganglion cells, but not as yet in photoreceptors [124]. BLRB is formed by the associated action of biliverdin reductase (BVRD) on another immediate HMOX1 product, biliverdin (BLVD), and has been shown to be the most efficacious antioxidant operating in the hydrophobic, membrane-bound compartments of the cell, much as GSH operates in cytoplasmic, largely aqueous subcellular zones [125]. Furthermore, just as a regenerative cycle exists for GSH, it has been proposed that BLVD, as the oxidized product of BLRB, is converted back to BLRB by enzymatic action of BVRD, using NADPH as a co-factor [125]. In our gene array results, we found positive FC for the DEG *Bvrda* in cells incubated with EPCD and 7kCHOL (Table S4), which would be appropriate for coordination between the enzyme products of this gene and of HMOX1. Although increased gene expression of *Nfe2l2* is not necessarily a prerequisite for amplified expression of targets whose regulatory sequences incorporate the ARE, there are “non-canonical,” genomic mechanisms for heightened expression and activity of *Nfe2l2*/Nrf2 that involve positive transcriptional regulation by the AHR-ARNDT heterodimer [126]. In this context, interestingly, exposure to 7kCHOL, a proven AHR ligand [127], but not EPCD, resulted in increased gene expression of *Nfe2l2* in our array (Supplementary Materials, Figure S3), and this could be the basis for the much higher intensity of HMOX1 immunoreactivity expression in the cultured 661W cells incubated with the former oxysterol vs. the latter.

Although signaling pathways leading to increased expression and activity—and therefore higher yield of the enzymatic products—of HMOX1 are considered cytoprotective in the short term, it has also been proposed that sustained activity of *Hmox1*/HMOX1 may have deleterious cellular effects that would result in cell death, as a result of accumulated toxic levels of the unconjugated heme catabolic products BLRB, CO, and especially ferrous ion (Fe^+2^), leading to increased oxidative stress and mitochondrial damage [128]. However, it should be noted that this pathological scenario specifically excludes changes in ferroportin (SLC40A1) expression [129]. On the contrary, the increased expression of *Slc40a1*—also a target of Nrf2 [130] —that we observed in oxysterol-treated 661W cells (Table S4) would be considered a homeostatic response to generation of Fe^+2^ from HMOX1-catalyzed heme breakdown [131] (Note that CHOL treatment also generated a FC >3 for the *Slc40a1* transcript in the absence of any changes in *Hmox1* (Supplemental Table S4)).

Induced expression of Hmox1 at the transcript and protein level is protective in vertebrate photoreceptor cells in response to different kinds of insults, both in vivo and in vitro, including ceramide challenge to 661W cells [132,133,134]. In neurons, up-regulation of HMOX1 has been identified as a protective response that mitigates stimuli that compromise cell viability [135]. However, direct evidence for any specific cell viability-enhancing effect of BLRB in ocular tissues has not been documented. Further elucidation of the contribution of elevated *Hmox1* expression to survival of photoreceptors and other CNS neurons in response to cellular stresses such as those caused by oxysterols awaits more detailed investigation, especially at the biochemical level, and additional information along these lines may direct future therapeutic approaches to SLOS, and to retinal and other neural degenerative diseases.

We validated oxysterol-induced up-regulation of DNA damage-inducible transcript 3 (*Ddit3*), the gene coding for CHOP (CCAAT/enhancer-binding protein homologous protein, also known as Growth arrest and DNA damage-inducible protein 153 (*Gadd153*)), by demonstrating pronounced, overwhelmingly nuclear, immunoreactivity for CHOP protein in oxysterol-treated 661W cells. CHOP expression is induced by a variety of forms of cell stress, and its up-regulation is a hallmark in particular of ER stress [136,137]. The immunocytochemical localization of CHOP in oxysterol-treated 661W cells is consistent with its function as a transcriptional (co-)factor, although there is evidence for activity of cytoplasmic CHOP as well [138]. 7kCHOL was previously shown to up-regulate CHOP expression, along with ER stress, in cultured aortic smooth muscle cells [29,139]; therefore, increased CHOP expression in oxysterol-treated samples is validation of the enrichment of this pathway. *Chop* is a target gene for ATF4, via activation of PERK—the predominant mode of CHOP transcriptional up-regulation—but also is up-regulated downstream from the other two arms of ER stress, by IRE1A and ATF6; several promoter regions are involved in ER stress-induced CHOP transcription [137].

Numerous DEGs highlighted in Figure 6 are transcriptional targets of CHOP, notably *Trib3*, *Ero1l*, and *Gadd34* [140,141,142]. CHOP expression was also consistent with up-regulation of *Atf4*, whose translated protein enters into a heterodimeric transcriptional factor complex with CHOP. As a heterodimer with either ATF4, or with CEBPB, CHOP regulates transcription of an extensive range of genes [143,144], and these, along with upstream modulators of *Ddit3*/CHOP transcription and function, illustrated in Supplemental Materials, Figure S5, are diagnostic of increased CHOP expression. Examples of DEGs shown to be CHOP targets include: *Chac1*, whose increased expression leads to depletion of glutathione and apoptosis [145]; *Fgf21*, a stress-responsive hormone that has been demonstrated to respond at the cellular level to ER stress [146,147]; *Nek6*, whose down-regulation in EPCD-treated samples is indicative of cell cycle arrest [148]; and *Pmaip1* and *Bbc3*, up-regulated in 661W cells by 7kCHOL incubation, whose translation products, NOXA and PUMA, respectively, are BH3-only BCL-2 family members that induce cell death by promoting mitochondrial permeability barrier breakdown [149,150] (Figure S5).

The operation of the cell cycle has been shown to be linked to neuronal cell death [151], although this process has largely been investigated using postmitotic neurons. In the context of 661W proliferation under initial conditions in the study described here, on the other hand, DNA damage can induce cells to interrupt cell division [152]. Cell cycle arrest is a pro-survival feature of the earlier stages of ER stress, which is assumed to be coordinated with selective control of protein translation and DNA synthesis [153]. *Ddit3* expression is associated with cell cycle arrest, in response to oxidative stress and DNA damage, and also downstream from PERK activation as an element of ER stress [154,155]. The association of CHOP with cell cycle arrest occurs in large part by expression of its target gene, cyclin-dependent kinase (CDK) inhibitor *Cdkn1a* (p21) [156,157]. p21 is a CDK inhibitor that mediates cell cycle arrest, especially in response to DNA damage and repair [158]. Cell cycle arrest stimulated by endogenous CDK inhibitors such as p21 has been noted as a mechanism for cells to optimize DNA repair in response to genotoxic stress [159]. Prevention of cell cycle reentry upon cell injury and degenerative stimuli is a mechanism that attenuates cell death of postmitotic cells such as neurons [151,160], whereas dysregulation of the cell cycle is implicated in neurodegenerations and neuronal cell death [161]. Transcriptional activation of *Cdkn1a* can be p53 independent [162], and we observed that EPCD treatment increased *Cdkn1a* expression, in contrast to down-regulation of this gene by CHOL (Figure S5).

Apart from ER stress, CHOP also has been shown to be a transcriptional target of BRCA1, in response to DNA damage [163], and *Brca1* was up-regulated in 661W cells by exposure to both EPCD and 7kCHOL (Figure S5). Phosphorylation of CHOP by MAPK12 (p38γ), the gene for which was up-regulated by EPCD, increases the transcriptional activity of CHOP [164]. The only DEG whose expression may not reflect enhanced CHOP levels that we detected was *Pcaf*, a DEG with negative FC induced by EPCD, and whose corresponding protein was shown to be a cofactor of ATF4 for positive regulation of *Ddit3* during amino acid deprivation [165]. Interestingly, expression of the cytoskeleton-associated protein ezrin was shown to down-regulate *Ddit3* and other stress-response genes (including *Trib3*) [166], and while *Ezr* was not a DEG in oxysterol-treated samples, its expression increased in CHOL-incubated cells (Figure S5). CHOP also modulates transcription of numerous genes directly involved in the autophagic process, including several that were up-regulated by exposure of 661W cells to the oxysterols tested here, namely *Atg12*, *Map1lc3b*, *Sqstm1*, *Nbr1*, and Atg8 homologs such as *Gabarapl1* [143,167] (Figure 11).

*Ddit3*/CHOP is central to cell death emanating from sustained ER stress, and preventing its expression protected photoreceptors from death associated with ER stress in animal models of retinal light damage and detachment [168,169]. Exogenous challenges, represented by exposure to oxysterols as applied in this study, can initiate homeostatic derangements, such as changes in cellular redox status, oxidative stress, and other metabolic disruptions, that negatively impact protein synthesis, folding, and downstream processing events. These imbalances are sensed and transduced in the ER, leading to the UPR and activation of the three canonical ER stress pathways [34], including the PERK cascade [170], which, owing to phosphorylation of EIF2α, at least temporarily ameliorates further potential loss of ER functional fidelity by inhibiting protein synthesis. Eventually sustained up-regulation and activation of CHOP by ATF4, also downstream from PERK activation [171], leads to transcription of genes reinstating active protein synthesis, including those coding for aminoacyl transfer RNA synthetases [144]; Supplemental Figure S6 shows the broad positive regulatory effect of oxysterol incubation on expression of these genes in 661W cells. The resumption of protein translation is due in large part to the dephosphorylation of EIF2α by GADD34, the product of *Ppp1r15a*, a target gene of the ATF4/CHOP homodimeric transcriptional complex, and whose up-regulation increases activity of the protein phosphatase PP1 [155]. Ultimately, the reducing environment of the ER required to maintain augmented protein folding, via coupled activity of protein disulfide isomerase and ERO1l (ERO1α) (Figure 6), generates hydrogen peroxide exceeding homeostatic levels [144,172], amplifying oxidative stress with repercussions throughout the cell, including DNA damage [173,174], that drive numerous modes of regulated cell death.

With the progression over time of events underlying the UPR and ER stress, the impact of *Ddit3* up-regulation and consequent expression of CHOP on the balance between cell death and survival is determined in part by the stoichiometric relations of CHOP and the binding partners with which it forms heterodimers. The more important of these transcription co-factors are ATF4, ATF3, and CEBPβ, all three of which were DEGs with positive FC in oxysterol-treated 661W cells (Figure 6 and Figure S5). The ATF4/CHOP heterodimer is transcriptionally active [144]—as is that of ATF4/ CEBPβ [175]—whereas the complexes of CHOP with ATF3 or CEBPβ are inactive or dominantly repressive [176,177]. It has been proposed that the sequence of events from primarily pro-survival responses to regulated cell death involves a progression of CHOP binding interactions from mostly ATF3 to predominantly ATF4 [178]. This scenario actually fits our experimental design of harvesting RNA from oxysterol-treated samples ahead of time points at which the majority of cells might have entered into the “execution” stage of cell death. Since CEBPβ expression has been shown to exert pro-survival effects [179,180], increased expression of CHOP has the potential to neutralize the activity of this transcription factor, increasing susceptibility of cells to cell death. However, because CEBPβ may be expressed as one or several of three isoforms with different activities [181], which are not individually discernible in our array results, a full account of the contributions of changes in expression of CHOP and its binding partners to oxysterol-induced 661W survival and death will await more detailed analysis at the transcriptional, translational, and functional levels.

There is evidence that p53 plays a role in the response of retinal cells to various forms of stress [182], and p53 regulates neuronal cell death in response to DNA damage [183]. Chop and p53 are potentially intertwined in a negative regulatory relation, in that *Chop* transcription may be negatively regulated by p53, and at the same time CHOP up-regulates *Mdm2*, whose protein product targets p53 for proteasomal degradation [163,184] (Supplemental Materials, Figure S5). Significant transcriptional regulation by p53 would be expected to have been dampened by the expression of SV40 T antigen as a result of immortalization of the 661W cell line [100]. In fact, EPCD and CHOL induced robust down-regulation of *Trp53* gene expression, which itself would not be affected by expressed SV40 T antigen (Figure S5). For additional discussion of SV40 T antigen, and p53 expression and activity in 661W cells, see Supplemental Materials: Supplemental Results and Discussion, Section S3.1. and Figure S7.

Tribbles homolog-3 (TRIB3) has been classified as a pseudokinase, lacking true kinase activity but capable of fulfilling important cellular functions, as a scaffold, adaptor, or docking protein in interactions with true kinases, ubiquitin ligases, and transcription factors, among other regulatory roles [185,186]. Elevated transcriptional expression of *Trib3* is correlated with ER stress-induced cell death, both in vivo and in vitro, notably as a response to treatment of cultured cells with tunicamycin or thapsigargin [187]. TRIB3 blocks phosphorylation and activation of Akt, leading to increased expression of the pro-apoptotic gene *Puma*, in a manner dependent on *Foxo3* expression [188]. On the other hand, TRIB3 has been shown to function in cell cycle checkpoint control and to protect DNA against double-strand breaks, consistent with a pro-cell survival role of this protein in the nucleus [189,190]. The balance between initial pro-survival and eventual emergence of pro-death gene and protein expression patterns documented for TRIB3 expression may be correlated with the effects of TRIB3 on gene activation and other macromolecular interactions, the most important example being transcriptional activation of *Trib3* by ATF4 and CHOP, leading eventually to repression of the *Atf4* and *Chop* genes themselves by TRIB3, thereby down-regulating its own expression in a negative feedback loop [140,191]. Interestingly, macrophages exposed to oxidized LDL, a component of which is 7kCHOL [30], display ATF4- and CHOP-dependent increased expression of *Trib3* [192]. Therefore, *Trib3* up-regulation may exert either a pro- or anti-apoptotic effect, depending on the relative stoichiometry of TRIB3 with its transcriptional regulators, which may govern the time course and end point of the stress response. Our results featuring high levels of expression of *Atf4* and *Chop* as well as *Trib3* in oxysterol-treated 661W cells may be a confirmation of our effort to capture a “snapshot” of gene expression when challenges to cellular integrity are being detected and addressed in cells whose viability still remains intact, while at the same time cell death-promoting pathways have been invoked and are accelerating, although short of the final outcome in most of the cells in the sample. The *Trib3* promoter also has a binding site for CEBPB, whose gene may be up-regulated by ER stress [193], driving *Trib3* transcription [140,194]. While incubation of 661W cells with either EPCD or 7kCHOL resulted in pronounced up-regulation of *Cebpb* (Figure S5), this transcription factor gene was down-regulated by CHOL.

TRIB3 up-regulates expression of the autophagy-associated gene and protein SQSTM1 (p62) [195], but concomitantly hinders the binding of SQSTM1 to ATG8 homologues [196], leading to a blockade of autophagic flux, defined here specifically as the progression of autophagosomes from formation to fusion with lysosomes. Increased TRIB3 levels also retard autophagic flux by preventing phosphorylation of MTORC1, which can promote neuronal cell death [197]. The finding of pronounced up-regulation of both *Sqstm1* and *Trib3* in EPCD- and 7kCHOL-treated 661W cells suggests a correlation between our oxysterol-treated cell culture model, and the demonstration of impaired autophagic flux in the RPE of AY9944-treated rats as well as cultured human SLOS-derived RPE [70].

HERPUD1 has been shown to be up-regulated in many cell types, including neurons, in response to ER stress [198,199], and it participates in the retrotranslocation of unfolded proteins from the ER to the proteasome as part of the ERAD process [200,201]. Increased expression of HERPUD1 in response to ER stress is considered neuroprotective, in part owing to its role in the degradation of ER membrane components, including TRP channels and IP3 receptors, that promote depletion of luminal ER Ca^2+^, leading to downstream effects associated with cell death [202,203,204]; among the indirect antiapoptotic effects of increased HERPUD1 expression, therefore, is suppression of Caspase-3 activation by Ca^2+^ [199]. Interestingly, reduced production of inositol phosphates, leading to impaired IP3 signaling, has been observed in cultured SLOS fibroblasts [205]. HERPUD1 also inhibits the degradation of GRP78 that occurs as a result of ER stress-induced N-arginylation and subsequent trafficking of GRP78 to cytoplasmic proteasomes [206]. Increased expression of HERPUD1 thereby maintains higher levels of GRP78 to fulfill a chaperone function in the ER during ER stress, specifically associated with HERPUD1 during ERAD [207]. In fact, retention of GRP78 in the ER may itself limit leakage of Ca^2+^ to the cytosol [208].

Integral membrane proteins, with HERPUD1 as a candidate example, are known to undergo proteolytic cleavage in either the Golgi apparatus or the ER, followed by trafficking to the nucleus to function as transcription factors; a well-known example of the former route is that of sterol regulatory element binding factors [209], while a regulated ubiquitin/proteasome dependent processing scenario has been proposed, among others, for ER to nucleus trafficking [210]. One possible nuclear role of HERPUD1 may be reflected in the finding that increased expression of its gene and protein has been correlated with protection from DNA damage [211]. HERPUD1 protein structure resembles that of RAD23A/B, in that all three contain domains that bind to XPC, which is involved in DNA damage repair [201], and HERPUD1 has an affinity for ubiquitin by virtue of its proteasome-interacting motif [212]. The fact that other ubiquitin-like domain proteins, such as RAD23B and also including Parkin, have been detected in the nucleus [213,214], and that other ER membrane ubiquitin ligases also reside in the nucleus, also a site for protein quality control [215], may be circumstantial evidence of similar proteasome-associated function(s) for HERPUD1.

There have been several previous transcriptomic and proteomic studies using in vitro or in vivo models of SLOS. Although detailed and statistically formal enrichment analyses such as presented here were not executed, the reported data included changes in selected genes, or in proteins with corresponding genes, that were of interest in the context of the present investigation. Korade et al. [216] cultivated mouse Neuro2a cells with knockdown of *Dhcr7* using CHOL-free medium, and confirmed abnormally elevated levels of 7DHC in the cells, although analysis for oxysterols was not included. *Phf10*, *Braf*, and *Cebpb* were identified as genes showing up-regulated expression compared to *Dhcr7*-expressing cells, in agreement with our results for oxysterol-treated cells in the context of DNA damage and repair [217,218] (Figure 15) and CHOP expression (Figure S5). In an Affymetrix gene array analysis of hindbrains from embryonic *Dhcr7* KO mice, one of the prominent functional classes of DEGs was Apoptosis [219]. Taking into consideration that the tissue samples in the cited study included non-neuronal cells, *Gadd45a* was up-regulated and *Bak1* was down-regulated. One notable difference between the findings of Waage-Baudet et al. [219] and ours was decreased expression of *Txnrd3*.

Our laboratory had previously reported that EPCD (being uniquely derived in vivo from 7DHC and therefore a signature SLOS oxysterol) and 7kCHOL were cytotoxic to 661W cells, a model cone photoreceptor cell line, with different potencies measured over an effective dose range, in like manner to earlier findings with a neuronal cell line [14,21]. In the absence of additional information about the formation of these two compounds in tissues and bodily fluids of SLOS patients, it is presumed that their different cytotoxic potential in model systems is based on their different molecular structures, most obviously, those of a sterol bearing a critically placed ketone vs. an endoperoxide functionality. One significant finding from analysis of the gene array results reported here is that although both oxysterols, EPCD and 7kCHOL, generate cellular stress at doses that lead up to eventual cell death, the effects of the two compounds differ in many details, both subtle and profound, as evinced in the gene enrichment and defined gene set results. Since EPCD is a molecule found only associated with SLOS pathology, it was important to isolate the effects of the two oxysterols on gene expression. However, it is acknowledged that 7DHC gives rise to a varied roster of by-products in living systems, each with varied toxicities and potencies [14], and, as this study makes clear, exerting different interactions with cellular biochemical pathways, processes, and organellar functions, as revealed by the gene expression results detailed here; therefore, the sum total of effects of this oxysterol mixture may differ from that of each molecule tested separately [220]. As can be seen from Figure 3, each of the three compounds tested on 661W cells generated, of the total DEGs for that molecule, between 41% to 53% genes not shared with either of the other two treatments; because the tally of DEGs was much higher for EPCD, its unique DEGs were also numerically greater. Major differences between EPCD and 7kCHOL treatments in terms of major enrichment categories were:

- Cellular responses to oxidative and nitrosative stresses,
- Regulation of cellular homeostasis,
- Apoptotic process,
- Cysteine endopeptidase activity,
- ERAD,
- PERK arm of ER stress,
- Autophagy,
- Mitophagy, and
- Cell cycle regulation.

In addition, even where both oxysterols demonstrated significant effects for an enrichment category, when signature and other affiliated genes for these pathways or processes were evaluated individually, additional important differences emerged between EPCD- and 7kCHOL-treated samples that would be expected to be due to, and to have bearing on, molecular mechanisms underlying “subprograms” for these categories. Two distinct and intriguing examples are, first, *Puma* and *Noxa* being up-regulated only by 7kCHOL as elements of what appeared to be caspase-independent cell death; and second, up-regulation of the cell cycle regulator gene *Cdkn1a* being limited to cells incubated with EPCD.

Many of the cell stress pathways demonstrated here to be affected in response to oxysterol exposure have also been documented in cell culture models of SLOS, including fibroblasts from SLOS patients, as well as a DHCR7-deficient cell line and neural stem cells from SLOS transgenic mice [221]. In these studies, accumulation of autophagosomes suggestive of impaired autophagic flux, dysfunctional mitochondria subject to mitophagy, and increased PINK1 expression were correlated with abnormally high cellular levels of 7DHC, but not with a CHOL deficit. These changes were attenuated by pretreatment of cells with antioxidants, suggesting that the pathways were functionally linked to oxidative stress [221]. It is tempting to speculate that 7DHC-derived oxysterols such as EPCD and 7kCHOL, which have been generated in cell-free systems by chemical oxidation of 7DHC [22], were responsible for the cellular dysfunctions noted in these cultured cell models of SLOS.

Our rationale for using CHOL as a control treatment and the method of its administration to 661W cells notwithstanding, incubation with this agent was recurrently found to induce DEGs in what could be interpreted as an anti-apoptotic/pro-cell survival pattern, often the opposite of what was generated for oxysterols, as shown in many of the enrichment results. In that respect it is interesting that CHOL replacement therapy has been proposed to treat SLOS patients [222,223]. The individual gene results for 661W cells incubated with CHOL were often exemplary of increased or decreased expression of DEGs with positive effects on cell viability, respectively. Some notable examples are CHOL-induced up-regulation of *Pink1*, and down-regulation of *Noxa*.

A different phenomenon is presented by the down-regulation of *Sesn2* by CHOL treatment, in contrast to its increased expression in 661W cells exposed to 7kCHOL (but not EPCD), as Sesn2 expression is associated with a protective, pro-survival response to several modes of stress; this may be an example of a hormetic effect [38]. In fact, there are several incidences in this study of genes nominally considered cytoprotective, either individually or as part of a pro-survival pathway or process, lacking apparent constitutive expression, that are up-regulated by one or more of the forms of stress described here, but whose sustained expression is either insufficient to prevent, or eventually contributes to, a switch from survival to cell death, with different modes of execution. Since our samples represent one time point, and one set of dosages, our data likely represent a single view within the transition stage of a dynamic process, such as described for just one ultimately cytotoxic pathway, ER stress [224].

The 661W cells employed for our gene array study represent a surrogate for retinal photoreceptor cells and also admittedly have certain limitations as an in vitro model of neurons, since at the time experimental treatments were initiated they were still proliferating. The gene expression findings reported here may be applicable in this respect to normally dividing neural precursors, and therefore our findings may provide some insight into the developmental aspects of SLOS pathophysiology. For example, ER stress and DNA damage and their downstream pathways, as well as stress and dysfunction affecting other selected subcellular organelles, have not previously been implicated as relevant molecular mechanisms that may underlie the SLOS neurological phenotype. Human neuronal cells that are postmitotic, whether they are cell lines or induced pluripotent stem cell-derived cells induced to differentiate, will be useful in future investigations of the effects of oxysterols, with relevance for the post-developmental course of SLOS as a neurological disease. However, there is at present no obvious strategy to program 661W cells to become postmitotic, differentiated cells with morphological and functional attributes more representative of fully differentiated photoreceptors.

The most obvious morphological shortcoming of the 661W cell line is the lack of elaboration of an outer segment (although these cells do express a cilium [225]), raising the question of whether this detracts from their being an apt model for the effects of oxidative stress on photoreceptors. Cone opsin proteins are expressed in 661W cells [21,226], but whether these function as visual chromophores is debatable in the absence of their incorporation into outer segment membranes. Although the polyunsaturated fatty acid docosahexaenoic acid (DHA; 22:6(n-3)) is concentrated in native rod and cone outer segments, 661W cells can indeed incorporate this polyunsaturated fatty acid and convert it to neuroprotectin D1 as a result of bright light treatment [227], actually promoting cell survival. However, this cell line also has been successfully utilized as an in vitro model of retinal light damage [228,229], supporting the proposed participation of photosensitizing targets in native photoreceptors proximal to the outer segment, for example in mitochondria, that respond to wavelengths in the blue range of visible light [101,230]. We propose that 661W cells may be adequate for study of photoreceptor cell-autonomous aspects of oxidative stress, distinct from those that require the participation of other retinal cell elements, such as RPE phagocytosis of shed distal portions of outer segment membranes [231,232,233]. The loss of outer segments in retinal degenerations may exacerbate oxidative stress because of decreased consumption of oxygen despite continued high levels of O_2_ in the outer retina [234]; for this reason, 661W cells might be an adequate model for demonstrating oxidative damage to similarly compromised photoreceptors. The concentration of mitochondria in the ellipsoid region predisposes native photoreceptors to oxidative stress, even in the absence of light stimulation [235,236].

While further advances in the technology of cell culture models of photoreceptors are expected to stimulate advances in the field, there is still potential to learn from 661W cells as a model of mammalian retinal photoreceptors. For example, our demonstration of DEGs associated with ER stress in oxysterol-challenged 661W cells complements recent findings that signature proteins and genes of this pathway are also operative in native visual cells subjected to light damage [237], and in those that express mutations causing retinal degenerations [238]. From an alternative point of view, insufficiencies in DNA damage response and repair stemming from dysregulation of genes underlying this pathway, such as those identified here, have been proposed to contribute to photoreceptor cell death in human retinitis pigmentosa [239]. Further, as suggested by Pan et al. [240], our results not only indicate that a single original insult may activate multiple avenues of cell death, but also underscore the crosstalk between them. The DEGs and gene expression patterns identified here may represent potential therapeutic targets, and it is expected that their elucidation will increase the understanding of photoreceptor degeneration and other retinal diseases, as a basis for future treatment and prevention.

## 4. Materials and Methods

### 4.1. Sterol and Oxysterol Reagents

Cholesterol (CHOL) and 7-ketocholesterol (7kCHOL) were purchased from Sigma-Aldrich Chemical Corp. (Saint Louis, MO, USA) and stored in solid form in source bottles at −20 °C, desiccated and protected from light. The oxysterol 5,9-endoperoxycholest-7-ene-3β,6α-diol (EPCD) was custom synthesized through directed oxidation of 7DHC and purified by previously described methods [22,97,241], and it was stored as a lyophilizate of known mass, in an argon atmosphere, desiccated, and protected from light at −80 °C, until just before use. Structures of CHOL and the two oxysterols used here, and of 7DHC from which the latter are known to be biologically derived, are given in Supplementary Materials Figure S1. 5 mM and 10 mM stocks of CHOL and EPCD, respectively, were made by dissolving these two compounds in dimethyl sulfoxide (DMSO; Sigma-Aldrich); after Ar purging, these solutions were stored desiccated and protected from light, at −20 °C, and used within one week.

### 4.2. Cell Culture Methods

661W cells were obtained from Dr. Muayyad Al-Ubaidi, University of Oklahoma Health Sciences Center, Oklahoma City, OK, USA, under terms of a Material Transfer Agreement. 661W cells are a simian virus 40 (SV40) large T antigen-immortalized cell line derived from a benign, induced retinal tumor isolated from an early postnatal mouse, and had been originally demonstrated to possess properties of cone photoreceptors [100,226]. Protocols and techniques for expanding initial 661W cultures and cryopreserving source stocks, for subculturing and adaptation to a more defined medium, and for the extensive characterization and authentication of these cells in our laboratory using genomic, transcriptomic, immunochemical/immuno-histochemical, and morphological analyses and criteria, have been previously provided in detail [21,242], and are briefly summarized here. Cells were received in passage 24, and underwent gradual adaptation during the next several passages from medium containing 10% fetal bovine serum (Atlanta Biological, Atlanta, GA) to growth medium with the following attributes (see also Table 3 in [242]): a greatly reduced serum component (0.2% (*v*/*v*) bovine calf serum (BCS; Lonza, Walkersville, MD, USA)); formulation using a basal medium composed of 1:1 DMEM:F-12 (both HEPES modification; Sigma-Aldrich); addition of supplements based on an updated recipe for Neurobasal medium [243,244]; and containing a HEPES-buffered saline extract of a bovine retinal homogenate as the only other undefined component, contributing added total protein for a final concentration of 6 mg per liter of complete medium. Cells were subcultured using Accutase [245] for enzymatic release from the growth substrate.

### 4.3. Experimental Treatments

Cells were seeded at passage 36 in poly-L-ornithine-coated 100-mm tissue culture plastic dishes (poly-L-ornithine from Sigma-Aldrich; dishes from Falcon, Billerica, MA, USA), at 100,000 cells/dish, in triplicate for each of the four treatments described below. Cultures underwent routine incubation at 36.5 °C, in a 6% CO_2_/90% humidified air. After 2 days, when the cultures had attained approximately 70% confluence, growth medium was replaced with 9 mL of incubation medium [21], a simplified medium composed of DMEM/F-12 with only the following substituents: BCS (0.2%), transferrin, hydrocortisone, non-essential amino acids, alanyl-glutamine, sodium pyruvate, triiodothyronine, glucose, fructose, and 2-hydroxy-3-[tris(hydroxymethyl)methylamino]-1-propanesulfonic acid (TAPSO) (Sources available in [242]). After overnight incubation in incubation medium, 1 mL per dish of one of the 10× working stocks of treatment agents was introduced, each dish was briefly swirled to effect mixing, and the dishes were incubated for time intervals noted below subsequent to harvesting of RNA.

Incubation periods, concentrations, and formulations for final treatments (n = 3 for each) were as follows:

(1) EPCD, 23 h (final concentration, 6 µM): A 10 mM source stock of EPCD in DMSO was thawed, vortexed, and initially diluted with vortexing, at 1:167, to 60 μM (10× the desired final concentration) in the required volume of incubation medium (in this and all following preparations the incubation medium had been briefly warmed and gassed in the cell culture incubator). The final DMSO concentration in the medium incubated with cells was 0.08% (*v*/*v*).
(2) 7kCHOL, 5 h (final concentration, 16 µM): 2 μL (10 μmol) aliquots of 5 mM 7kCHOL in absolute ethanol were dried in amber Eppendorf tubes and stored under Ar at −20 °C until further use. Source stock: To the 7kCHOL was added 1 mL of a 1:9 (*v*/*v*) mixture of absolute EtOH and 45% (*w*/*v*) hydroxypropyl-β-cyclodextrin (hpβCD; Sigma-Aldrich) in water [246]; this mixture was sealed with Ar, protected from light, and vortexed periodically at room temperature for 1 h, when a clear solution was formed. This 10 mM aqueous stock in complexation with hpβCD was diluted with vortexing, at 1:62.5, to 160 μM (10× the desired final concentration) in incubation medium. The final hpβCD concentration exposed to cells was 720 µg/mL, and the nominal final ethanol concentration was 0.0016%.
(3) CHOL, 23 h (final concentration, 8 µM): A 5 mM stock in DMSO was thawed, vortexed, and diluted with vortexing, at 1:62.5 to 80 μM (10× the desired final concentration) in the required volume of incubation medium. The final DMSO concentration exposed to cells was 0.16%.
(4) hpβCD vehicle control (VC), 24 h: The hpβCD/EtOH-only stock otherwise used to formulate the 7kCHOL treatment ((2), above) was diluted to a 10× stock in incubation medium by 1:62.5 (equivalently to the 7kCHOL stock), for final concentrations with cells of 720 µg/mL hpβCD and 0.0016% EtOH.

The rationale for the different exposure times was based on a strategy of capturing the status of the transcriptome when either of the two ultimately lethal oxysterol treatments would have initiated the postulated regulated cell death program(s) and also might have caused associated responses—some even pro-survival and protective—to be brought into effect, but before an overwhelming proportion of cells had undergone the final stages of cell death, i.e., at the point where concomitant degradation of cell molecular machinery and substructure supporting mRNA integrity had already largely ensued. With this goal in mind, pilot studies were carried out with several doses of EPCD and 7kCHOL, which were monitored morphologically over time (using an inverted microscope), to determine optimal treatment interval parameters for both oxysterols. It was also confirmed that 23–24 h of incubation with either hpβCD VC or the CHOL treatments did not result in loss of cell viability (Figure 1A,D). We had previously demonstrated these non-lethal effects using a quantitative dual reagent cell viability assay for even higher doses of either hpβCD or CHOL [21]. It should be noted that based on a time course study using two combined quantitative assays, we already had observed that the time frame for complete loss of viability of 7kCHOL-treated cells could be much less than 24 h (see Supplementary Results in [21]). In contrast, for EPCD, the morphological changes leading up to universal death of 661W cells appeared to be gradual throughout the area of the culture dish, despite the greater potency of EPCD vs. 7kCHOL, possibly reflecting differences based on molecular structure influencing the cellular mechanisms invoked (cf. Figure 1B,C, respectively).

### 4.4. Isolation and Quality Assessment of Total RNA

At the incubation times noted above for each treatment, incubation media were aspirated from each replicate dish, and cells were lysed in the dish using the buffer (containing 0.5% NP-40 equivalent as the detergent) supplied with the RNeasy^®^ Plus Minikit (Qiagen, Germantown, MD, USA) according to the manufacturer’s protocol. Lysates were collected using a scraper and filtered through a Qiashredder spin column (Qiagen). Processing of the samples progressed further as instructed using the kit materials, and the final preparations of total RNA in RNAse-free water were stored at −80 °C awaiting the next steps. RNA yields and initial working stock concentrations for each sample (Supplementary Materials Table S5) were calculated using the ratio of 260/280 nm absorbance values (= 2.1 for all samples), measured by means of the microdrop technique in a Synergy-HT plate reader (BioTek, Winooski, VT, USA). RNA integrity for each sample was assessed in agarose gels using a Bioanalyzer (Model 2100, Agilent, Santa Clara, CA, USA), with all sample RIN values = 10 (Not shown) [247].

RNA degradation plots were generated using arrayQuality Metrics [248], utilizing preprocessed data (subjected to background correction and normalization). The individual arrays exhibited virtually parallel traces with no obvious outliers, and all lines had overall slopes (5′ to 3′ in the *x* axis) between 3.0 and 3.3 (*results not shown*), indicating excellent preservation of integrity encompassing samples both within and across treatment groups. The integrity and degradation determinations aided in ruling out DEG results being ascribed to differences in sample handling and processing, as well as inconsistencies in probe chip performance or quality control [249].

### 4.5. Processing of RNA Samples, Reading of Hybridized Arrays, and Processing of Raw Data

Mouse Genome 430 2.0 arrays (Affymetrix, Santa Clara, CA, USA) were used for hybridization assays, which were performed at the Next-Generation Sequencing and Expression Core Facility, University at Buffalo (Buffalo, NY, USA). Final sample preparation/amplification, biotin labeling, and fragmentation of complementary RNA were also carried out at the Next-Generation facility using standard supporting equipment and kits, following Affymetrix specifications and protocols, to generate targets, for each replicate sample representing all four treatment groups to be analyzed; these included the WT Expression Kit (Ambion, Carlsbad, CA, USA), and the Flash Tag Biotin HSR RNA Labeling Kit (Affymetrix). Following incubation with streptavidin-phycoerythrin, hybridized chips were read in a Model 3000 GeneChip^®^ high resolution array scanner (Affymetrix), and raw intensity data in *.cel files were generated; normalization, background correction, “housekeeping” gene, and hybridization control values were also incorporated in each array.

Bioconductor (with R statistical computing environment; R Core Team, 2014), v.2.13 (www.bioconductor.org) [250], in particular the affy package [251], was then used to process and convert raw data into triplicate relative gene expression values corresponding to each array probe. Arrays were normalized using the *expresso* function with loss correction and perfect match-only median polish summarization. Empirical Bayes moderated t-tests were then utilized to compare experimental conditions, which permitted the computation of mean “fold change” (FC) values for EPCD, 7kCHOL, and CHOL treatments, each vs. VC. Corresponding q-values (false discovery rate adjusted *p*-values, or AdjP [25]) for each gene in these comparative data sets were also calculated. Except where noted, DEGs were selected by their |FC| (positive or negative absolute value) being ≥ 1.5, and with AdjP ≤ 0.0010. DEG sets underwent annotation/enrichment analysis, with the implementation of the online software program DAVID, version 6.8 (https://david.ncifcrf.gov/) [31,32], to identify differentially regulated pathways, processes, and functional components.

Raw microarray data (.cel) files and associated MIAME information are available in the ArrayExpress database (http://www.ebi.ac.uk/arrayexpress) under accession number E-MTAB-10055.

### 4.6. Immunofluorescence Detection of Proteins Corresponding to Selected DEGs

#### 4.6.1. Preparation and Treatments of 661W Cells for Confocal Microscopy

Chamberslides (4-well), made with surface-modified glass as per Kleinfield et al. [252] (Lab-Tek System II, Thermo Fisher Scientific, Waltham, MA), were first treated with poly-L-ornithine (4 µg/cm^2^; working stock in sterile water, diluted from 0.01% (*w*/*v*) source stock obtained from Sigma-Aldrich) [242], and then 661W cells between passages 40 and 50 were seeded at 10,000 cells/well, arranged in four treatment designations: EPCD (6, 8, or 10 µM); DMSO VC (0.1 % (*v*/*v*), matching the final dilution from EPCD stock); 7kCHOL (20 or 25 µM); and hpβCD VC (0.009% (*w*/*v*)), matching the lower dilution from 7kCHOL working stock). Stocks and dilutions to 10× desired final concentrations of experimental agents were created as for the gene array samples, above. After seeding, cells were maintained for 1–2 d in a growth medium volume of 800 µL until they reached approximately 75% confluence, at which point 500 µL of the total medium volume was exchanged for 420 µL incubation medium (*see above*). On the next morning, following a further overnight incubation, cells received experimental treatments, by addition of 80 µL of 10× working stocks, and the cultures were monitored by microscope over a period of 4 to 24 h (see Section 2.3., and legend to Figure 1 for details) to evaluate morphological changes, assumed to be associated with cellular responses to the specific treatments and, for oxysterols, the expected progression towards eventual cell death (also as inferred from the previous gene expression analysis); the evaluation of these cellular modulations were based on criteria described previously [21], namely: retraction of neurites, elongation to bipolarity, cell rounding, and incipient detachment and loss of phase-refractivity, i.e., similar to the distribution of cell morphologies noted at the time points attained at the time of preparation of the RNA samples. Documentation of the appearance of the cultures at the time of fixation for immunohistochemical studies, and also of parallel cultures at later time points to confirm their ultimate viability status, was accomplished using digital photography with phase-contrast optics on an Axiovert 25 CFL inverted microscope (Zeiss, Thornwood, NY, USA) equipped with an Evolution^TM^ MP digital CCD 5.0 MP camera (MediaCybernetics, Rockville, MD, USA).

#### 4.6.2. Fixation of Treated 661W Cells

After suitable incubation periods for experimental treatments, cells were then fixed in situ by either of the following two protocols:

##### Formaldehyde Fixation

Immediately upon removal from the cell culture incubator, 200 µL of treatment medium was removed from each Chamberslide well, to be replaced with 200 µL of fixative (4% formaldehyde (diluted from an approximately 37% (*w*/*v*) stock solution of molecular biology grade formaldehyde (Sigma-Aldrich) in modified Hanks’ balanced salt solution (BSS), containing Ca^2+^ and Mg^2+^, buffered with HEPES-KOH (pH 7.5)) [253], and fixation commenced under ambient conditions for 10 min. The fixative/medium mixture was then exchanged with 1 mL of formaldehyde fixative (as above) only, and the Chamberslides were placed on ice for another 10 min. After three 20 min rinses with ice-cold phosphate-buffered saline (PBS), the slide chambers containing the final rinse solution were sealed against evaporation with Parafilm^®^ (Bemis, Neenah, WI, USA) and stored at 4 °C at least overnight, but no longer than one week, pending further processing. At the beginning of the immunofluorescent labeling protocol, any potentially exposed reactive aldehyde groups in the fixed cells were quenched by sequential treatments with, first, 100 mM glycine (in PBS, pH 8.0; Sigma-Aldrich) and then sodium cyanoborohydride [254] (from a stock in 0.1 N NaOH, diluted to a working concentration of 50 mM in PBS containing 10% (*v*/*v*) methanol; Sigma-Aldrich). Each quenching step was 15 min, with several intervening PBS rinses between these two steps, and two final rinses before blocking (see below).

##### Methacarn Fixation

Following a brief, gentle rinse of the cultured cells in RT Hanks’ BSS containing Ca^2+^ and Mg^2+^, the BSS was shaken from the Chamberslides, and the plastic chambers were removed from their mounting using the tool provided by the manufacturer. The wet glass slides, with cell growth areas still separated by gaskets, were immediately inserted into glass slide jars containing ice-cold methacarn (HPLC grade methanol:HPLC grade chloroform:glacial acetic acid (all from Sigma-Aldrich) in the volumetric proportions 60:30:10) [255]. After 10 min, slides were briefly dipped in ice-cold absolute EtOH, and then transferred for 15 min to ice-cold denatured EtOH (Histoprep, ThermoFisher, Hampton, NH, USA). Slides were stored in 70% denatured EtOH at 4 °C until further processing. Just before blocking, slides underwent equilibration to PBS via a graded EtOH:PBS solution series.

#### 4.6.3. Immunohistochemistry

Slides were blocked for one h, at RT, in 25 mM Tris-HCl buffer (pH 7.6) containing 220 mM NaCl (buffer reagents from Sigma-Aldrich); 0.2% bovine serum albumin (BSA; radioimmunoassay grade, Sigma-Aldrich); 0.5% fish skin gelatin (Sigma-Aldrich); 0.01% avidin (Sigma-Aldrich); and 5% (*v*/*v*) normal goat or horse serum (Vector Laboratories, Burlingame, CA, USA) to match the species of the secondary antibody to be employed, plus either 0.05% saponin (sapogenin-free; Sigma-Aldrich) for immunohistochemical localization of intracellular membrane-bound antigens [256], or 0.25% Triton X-100 (Sigma-Aldrich) for other antigens. All immunochemical incubations were performed in a humidified chamber. Chamberslide gaskets permitted the spatial isolation of different immunochemical treatments on one slide. Primary antibody incubations were carried out overnight at 4 °C, using antibody diluent consisting of Tris-saline buffer containing 0.1% BSA, 0.2% (*v*/*v*) Tween-20 (Sigma-Aldrich), and 0.002% biotin (Sigma-Aldrich). Sources, properties, and dilutions of primary antibodies are provided in Table 2. After a rapid initial rinse in Tris-saline buffer with 0.02% Tween-20 added, followed by a 10 min rinse in the same solution, slides were washed twice in Tris-saline buffer without detergent, 10 min each, before the next immunochemical step. Secondary antibody treatments were at RT for 1.5 h, with either biotinylated goat anti-rabbit IgG (with spacer) or biotinylated horse anti-mouse IgG (the latter for samples probed with anti-CHOP; both secondary antibodies were obtained from Jackson ImmunoResearch Laboratories, West Grove, PA) at 5 µg/mL, in Tris-buffered saline plus Tween-20, followed by the rinse regimen as above. Final incubations for all samples were with streptavidin-AlexaFluor 488 conjugate (Molecular Probes, Eugene, OR) at 8 µg/mL for 1 h at RT. After rinses equivalent to those following primary antibody, slides were equilibrated with PBS and incubated for 5 min with a 0.0001% (*w*/*v*) solution of 4′,6-diamido-2-phenylindole (DAPI; Biolegend, San Diego, CA, USA) in PBS. Following PBS rinses, slides were coverslipped with a 1:1 (*v*/*v*) mixture of Vectashield (Vector Laboratories) and PIPES-buffered Fluorogel (Electron Microscopy Sciences, Hatfield, PA, USA), and were stored refrigerated and protected from light for up to one week until examination using a laser scanning confocal microscope (TCS SPE II, fitted with a DMI4000 inverted microscope, and with AF6000 software, Leica Biosystems, Bannockburn, IL, USA). Laser lines at 405 and 488 nm were employed for detection of DAPI and AlexaFluor 488 fluorescence, respectively, with laser power, gain, and offset optimized to minimize background fluorescence, and appropriate excitation/emission windows to maximize signal while eliminating overlap and crosstalk. Frame averaging was set at 2. Digital images were captured using a 63× oil-immersion objective lens by sequential scanning, and saved as maximum projections of z-stacks (combined serial optical sections scanned in the x–y plane). All final immunofluorescent images represent equal numbers of optical sections, with equivalent pre- and post-capture adjustment of dynamic range. Focused digital images of matching fields for each fluorescence image using differential interference contrast (DIC) were also acquired.

### 4.7. Gene Enrichment and Other Analyses

Curations for analysis of DEGs were based on literature searches in Medline via either Pubmed (https://pubmed.ncbi.nlm.nih.gov/) or Ovid (Norwood, MA, USA). For enrichment analysis using the DAVID Analysis Wizard [31,32], the following strategy was employed: The analysis was initiated by entering and submitting the list, in the upload menu, of either positive or negative FC DEGs for a selected treatment identified as “OFFICIAL GENE SYMBOL” and “Gene List.” In the Gene List Manager tab, “*Mus musculus*” was highlighted for “Species,” “List Manager” was used to optionally assign a name to the analysis, and from the “Background” tab, “Mouse Genome 430 2 Array” was selected in the “Affymetrix 3′ IVT Background” field. Next, the Functional Annotation Chart was chosen, and Categories were opened for view by selecting “Gene Ontology” and a specific category (e.g., BP (Biological Processes) All) (or alternatively, “Pathways--KEGG”) from within “Annotation Summary Results.” By selecting “Chart,” results for each GO (or KEGG) term were displayed in a new window, which offered a choice of Options revisions to be chosen for statistical criteria and threshold parameters. For our enrichment analysis, we routinely selected Count = 2; Ease = 0.2 (which permitted inclusion of terms having *p*-values of greater than 0.05), display of “Fold Enrichment,” and application of Fisher’s Exact Test.

## 5. Conclusions

In a transcriptomic study of the cone photoreceptor-derived cell line 661W treated with cytotoxic doses of EPCD and 7kCHOL, two structurally different oxysterols (the former being specific to SLOS), we found enrichment of DEGs associated with ER stress/ERAD, DNA damage and repair, oxidative stress, autophagy (including mitophagy), and the mTORC1/2 pathways. In contrast, the results for CHOL treatment were consistent with its inclusion as a non-cytotoxic control. Enrichment analysis was validated by expression patterns of signature genes in these categories, and immunohistochemical detection of selected up-regulated translation products. These included CHOP, a canonical marker for ER stress, which was correlated with DEGs involved in the UPR, cell cycle arrest, and cell death. Simultaneous up-regulation of Hmox1 transcripts and immunoreactivity in oxysterol-treated 661W cells suggested increased antioxidant capacity in response to oxysterol-induced stress. The overall pattern of gene expression was consistent with a transcriptional “snapshot” at a time point when competing cell survival and cell death pathways were operative, before the latter became ascendant.

Our results support the hypothesis that generation of cytotoxic oxysterols contributes to the pathophysiology of SLOS, and the novel association of 7DHC-derived oxysterols with ER stress and DNA damage augments previously documented progress towards elucidating the molecular mechanisms underlying this disease. The transcriptomic results of the experimental treatments described here using a cone photoreceptor-derived cell line provide further insight regarding cell signaling pathways involved in the onset and course of retinal degenerations. It is becoming clear that regardless of the original insult or stressor, there are common cellular processes into which the various neurodegenerative diseases dovetail, with extensive crosstalk between subcellular stress responses. Because it incorporates exposure to endogenously derived small molecules as the initiating step, our in vitro model potentially may be extended to additional neural cell systems, with potential for isolating and characterizing therapeutic targets for treating neurodegenerative diseases.

## Figures and Tables

**Table 1:** Genes differentially regulated by all three sterols.

EPCD	7kCHOL	CHOL	Number of Genes
↑	↑	↑	49
↑	↑	↓	15
↓	↑	↑	7
↓	↑	↓	15
↓	↓	↑	5
↓	↓	↓	120

Upward arrows (↑), DEGS with positive FC values; downward arrows (↓), DEGs with negative FC values.

**Table 2:** Antibodies used for Immunofluorescence Confocal Microscopy.

Dilution	Cat. No.	Source	Host Species ^1^	FC, Array vs. VC		Subcellular Localization	Pathways	Symbol	Protein
				7kCHOL	EPCD				
2 μg/mL	13243	Abcam	R PC	5.646	10.743	ER, caveolae, nucleus	Oxidative stress	HMOX1	Heme oxygenase-1
1:1000	2895 (L63F7)	Cell Signaling	M MC	5.296	7.261	Nucleus	ER stress; apoptosis	CHOP	DNA damage-inducible transcript 3
2 μg/mL	R12-2387	Assay Biotech	R PC	7.056	8.424	Nucleus, cytoplasm	ER stress	TRIB3	Tribbles homolg-3
2 μg/mL	PA5-29469	Thermo Scientific	R PC	2.561	4.061	ER, nucleus	ER stress	HERPUD1	Homocysteine ER stress-inducible

^1^ R, rabbit; M, mouse; PC, polyclonal; MC, monoclonal.

## Data Availability

The array data .cel files, as well as supporting MIAME information, are openly available in the ArrayExpress database (http://www.ebi.ac.uk/arrayexpress) under accession number E-MTAB-10055.

## Supplementary Materials

Supplementary text (https://www.mdpi.com/1422-0067/22/5/2339/s1) with Supplemental Figures S1–S7, and Supplemental Tables S1–S5 (Word document); Supplemental Table S1A,B: DEGs with highest and lowest -FC (Excel document); Supplemental Table S2A–C: Positively and negatively expressed DEGs by treatment (Excel document).

## Abbreviations

Full names corresponding to gene symbols, referred to in the text, including synonyms for gene symbols, can be found at: www.genecards.org.

7DHC	7-dehydrocholesterol
7kCHOL	7-ketocholesterol
AdjP	adjusted *p*-value
ARE	antioxidant response element
BCS	bovine calf serum
BLRB	bilirubin
BLVD	biliverdin
BVRD	biliverdin reductase
BSA	bovine serum albumin
BSS	balanced salt solution
CDK	cyclin-dependent kinase
CHOL	cholesterol
CHOP	C/EBP homologous protein (also known as: Ddit3; Gadd 153)
CNS	central nervous system
CO	carbon monoxide
DAPI	4′,6-diamido-2-phenylindole
DAVID	database for annotation, visualization and integrated discovery
DEG	differentially expressed gene
DHCR7	sterol Δ7-reductase
DIC	differential interference contrast
DMSO	dimethyl sulfoxide
EPCD	5,9-endoperoxycholest-7-ene-3β,6α-diol
ER	endoplasmic reticulum
ERAD	endoplasmic reticulum-associated protein degradation
FC	fold change
GO	gene ontology
GSH	reduced glutathione
Herpud1	homocysteine-responsive ER protein with ubiquitin-like domain 1
Hmox1	heme oxygenase-1; HO-1
hpβCD	hydroxypropyl-beta-cyclodextrin
PBS	phosphate-buffered saline
PCA	principal component analysis
RIN	RNA integrity score
RPE	retinal pigment epithelium
SLOS	Smith–Lemli–Opitz syndrome
SV40	simian virus 40
Trib3	tribbles homologue 3; Trb3
UPR	unfolded protein response
VC	vehicle control
WAG	weighted average difference
