# Supplementary material for: Transcriptomic Changes Associated with Loss of Cell Viability Induced by Oxysterol Treatment of a Retinal Photoreceptor-Derived Cell Line: An In Vitro Model of Smith–Lemli–Opitz Syndrome
Source: Int J Mol Sci. 2021 Feb 26;22(5):2339. doi: 10.3390/ijms22052339 (PMC7956713; doi:10.3390/ijms22052339)
Supplement: Supplementary file 1 [file ijms-22-02339-s001.zip › Pfeffer et al_ijms-1110907_Supplementary Materials_rerevision_2-24-21.docx]

Pfeffer et al., **Supplementary Materials**

**S.4. Materials and Methods**

*S.4.1. Sterol and Oxysterol Reagents*


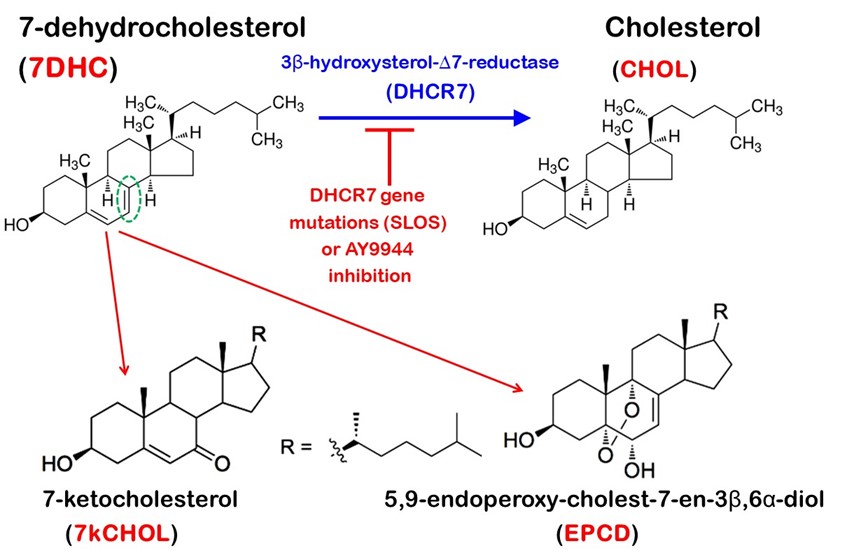


**Figure S1:** Structures and pathways for 7-dehydrocholesterol (DHC), and for 7DHC-derived oxysterols and cholesterol (CHOL) used for 661W cell culture incubations before harvesting total RNA.

**S.2. Supplementary Results**

*S.2.2. Gene Enrichment Analysis*

S.2.2.3. Oxidative Stress

Oxidative stress includes the effects of reactive nitrogen species, and **Figure S2, Columns A** shows that only EPCD treatment resulted in DEGs categorized for reactive nitrogen species metabolism and for response to nitrosative stress (**Figure S2 (B)**). Keeping in mind that EPCD is an endoperoxide (*Supplementary Materials* **Figure S1**), the suggestion that both of the oxysterols employed in this study exert some of their gene regulatory effects via peroxide, directly or as metabolic product(s), may be seen from **Figure S2 (C)**. Finally, CHOL itself demonstrated potential antioxidant activity by stimulating downregulation of four genes annotated for the GO: term “Positive regulation of superoxide anion generation,” namely *Cxcl1, Cyba, Edn1, and F2rl1* (**Figure S2 (D);** individual gene results not shown).


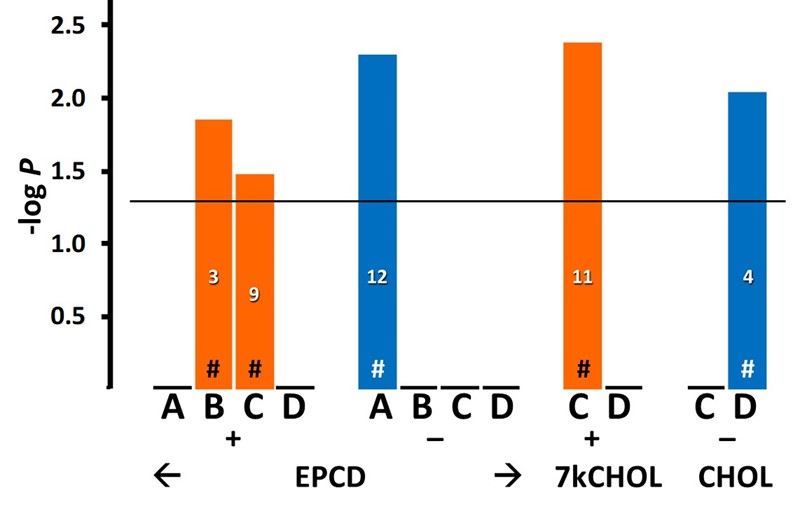


**Figure S2:** Gene Enrichment analysis using the following GO: terms: **A**, Reactive nitrogen species, metabolic process; **B**, Cell response to nitrosative stress; **C**, Cellular response to hydrogen peroxide; **D**, Positive regulation of superoxide anion generation. Note that for (D), four genes are down-regulated by CHOL; they are identified from the GO: term list as *Cxcl1*, *Cyba*, *Edn1*, and *F2rl1*.

S.2.2.3.1. Antioxidant Genes

In response to changes in intracellular redox balance, and to chemical stress induced by xenobiotics, heavy metals, and other electrophilic and prooxidative agents, genes coding for neuroprotective enzymes classified as Phase II antioxidants are upregulated by the transcription factor NRF2, which binds to antioxidant response elements of target genes [1]. At the most basic mechanistic level, NRF2—whose gene (*Nfe2l2*) itself has an ARE—is released from its cytoplasmic sequestration with KEAP1 as a result of chemical modification of the latter by the forementioned chemical challenges [2]. Phosphorylation by PERK can also dissociate NRF2 from KEAP1 [3], linking this transcriptional process to ER stress (**Figure 6**). *Nfe2l2* was upregulated by 7kCHOL, while its change in expression induced by EPCD did not meet the threshold for a differentially expressed gene (DEG) (**Figure S3**); however, it should be pointed out that the immediate transcription of Phase II enzymes does not depend on transcription of the gene coding for NRF2 itself. **Figure S3** shows the effects of oxysterols and CHOL on DEGs for antioxidant enzymes, most of which are NRF2 targets. The curated list includes glutathione peroxidases [4]; glutathione S-transferases [5]; and thiol redox modulators glutaredoxin and peroxiredoxin 6, plus genes for thioredoxin-associated enzymes TXNRD3 and TXNIP (the latter shown in **Figure S4**) [6]. *Txnrd3*, coding for a thioredoxin regenerating enzyme, is not directly transcribed by NRF2, but *Klf9*, the gene for a potent transcriptional suppressor of *Txnrd3*, is a NRF2 target [7]; although *Klf9* was a DEG with positive fold change (FC) for all three experimental treatments, its magnitude of FC induced by 7kCHOL was approximately triple that for EPCD (**Figure S3**). Zucker et al. [7] have pointed out that once ROS challenge exceeds a critical threshold, the subsequent elevated expression of KLF9 itself down-regulates transcription of *Txnrd3*, in a feed-forward pathway of cytotoxic oxidative stress. Expression changes of *Txnrd3* were only observed with EPCD treatment (**Figure S3**), but the pronounced effects of 7kCHOL on *Klf9* transcription may reflect a dysregulation in the oxidative stress response that is suggested in the enrichment results illustrated in **Figure 9**. Sulfiredoxin, whose gene (*Srxn1*) was upregulated by EPCD incubation of 661W cells (**Figure S3**), is an oxidoreductase that is translocated into mitochondria where it regenerates the activity of hyperoxidized peroxiredoxin 3 under conditions of excess H_2_O_2_ production [8]. Interestingly, redox homeostasis is maintained by the proteolytic disposal in mitochondria of SRXN1 by LONP1, product of a DEG with positive FC in oxysterol-treated samples [8] (Not shown).


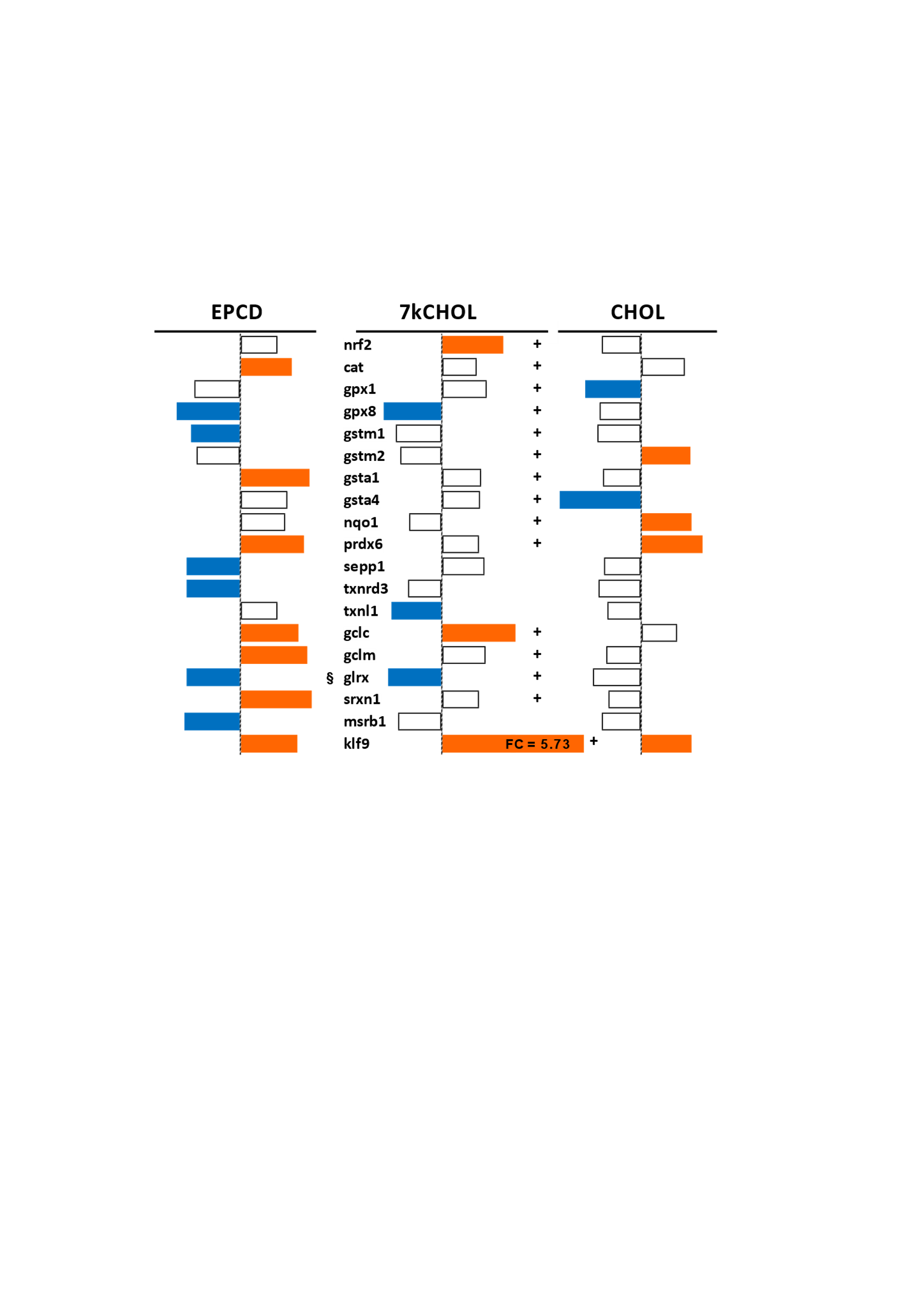


**Figure S3**: Array results for specific genes for proteins involved in antioxidant and redox regulating activities. Genes shown to have AREs, and/or that are transcriptionally activated by Nrf2, are indicated with a plus (+). §, different adjusted P-value (AdjP) ≤ 0.001.

It was anticipated that a pattern of expression for the DEGs shown in **Figure S3** would be discernible— but this was not the case, as many of the genes whose translation products fulfill antioxidant, and therefore presumably cytoprotective functions, were in fact down-regulated and there was little correlation with known NRF2 expression (e.g., genes upregulated by CHOL treatment). In the context of oxysterol-induced cell death, it can only be speculated to what degree increased or decreased expression of ostensibly cytoprotective antioxidant macromolecules plays a role in the final outcome. It is certainly possible that other regulatory modulators, such as KLF9 (above), could have exerted effects on expression of the genes portrayed in **Figure S3**. Furthermore, it has been suggested that under some conditions of sustained redox challenge, dysregulation of antioxidant expression may occur, which may have deleterious consequences [9,10].

S.2.2.4.Mitophagy

One mechanism that cells, including neurons, employ when confronted with damaged or malfunctioning mitochondria is to engage in autophagic disposal of these organelles, or mitophagy [11]. Array results for selected DEGs associated with this process, either directly or indirectly, are shown in **Figure S4**. In the context of the cell treatments, the overall trend of up-regulation for most of the genes depicted suggests an increase in mitophagy, in response to stress, that would enhance survival. Not only were most of the DEGs upregulated by incubation with either of the two oxysterols, but also by CHOL; at the same time, the patterns were different across the three treatments, with only four overlaps between the oxysterols, and two between CHOL and one or both oxysterols (**Figure S4**). However, the significant down-regulation for at least two genes, *Phb* and *Ugcg* (also shown to participate in mitochondrial quality control and sphingolipid metabolism, respectively [12,13]), may also be indicative of a dysregulation of mitophagy that could predispose the oxysterol-treated cells to an inadequate response to the other challenges to cellular homeostasis. It is also of interest that only CHOL incubation led to significant up-regulation of *Pink1* (**Figure S4**), which plays a central role in mitochondrial quality surveillance and the molecular recognition of damaged and malfunctioning mitochondria by mitophagic machinery [14], and whose expression might have a positive homeostatic influence in 661W cells whose viability was not compromised.


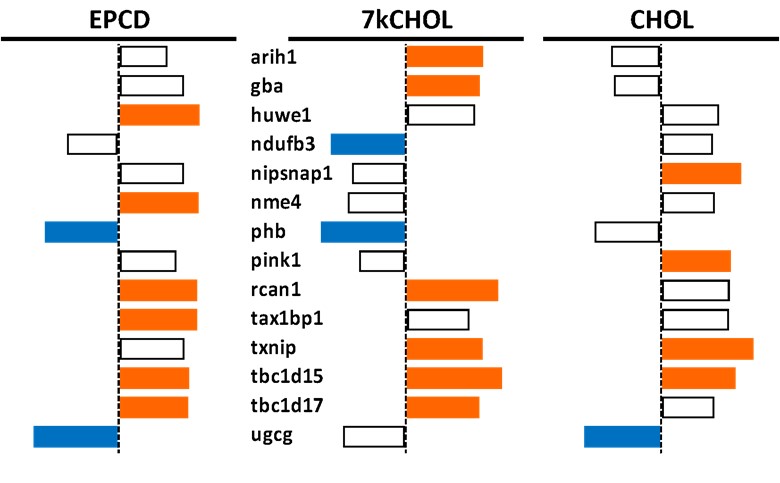


**Figure S4:** Array results for specific genes associated with mitophagy.

S.2.3.3.1. ER stress- and CHOP-Associated Genes


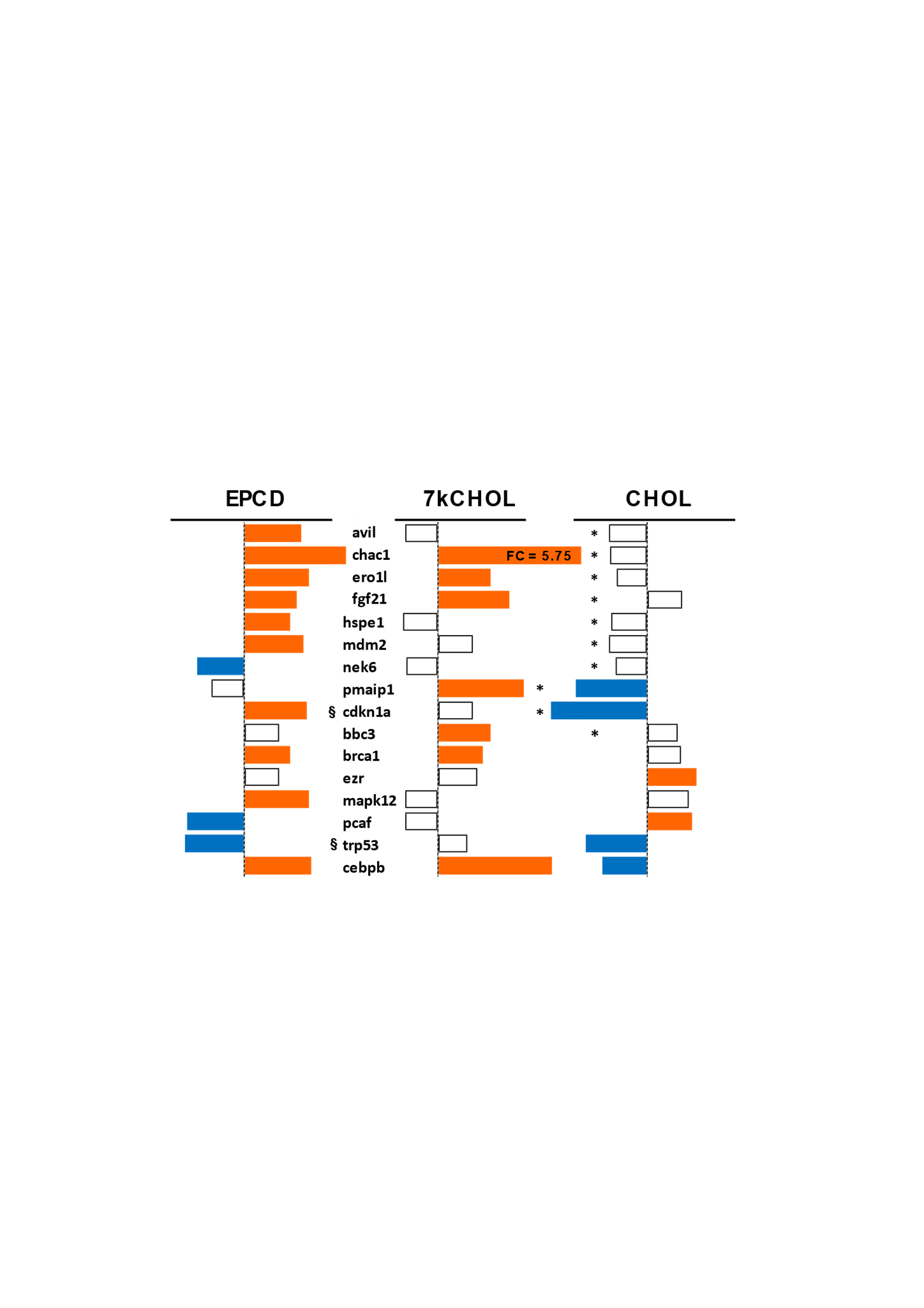


**Figure S5:** Array results for genes associated with CHOP target genes (*), and genes regulating CHOP expression and activity. §, different AdjP ≤ 0.001.


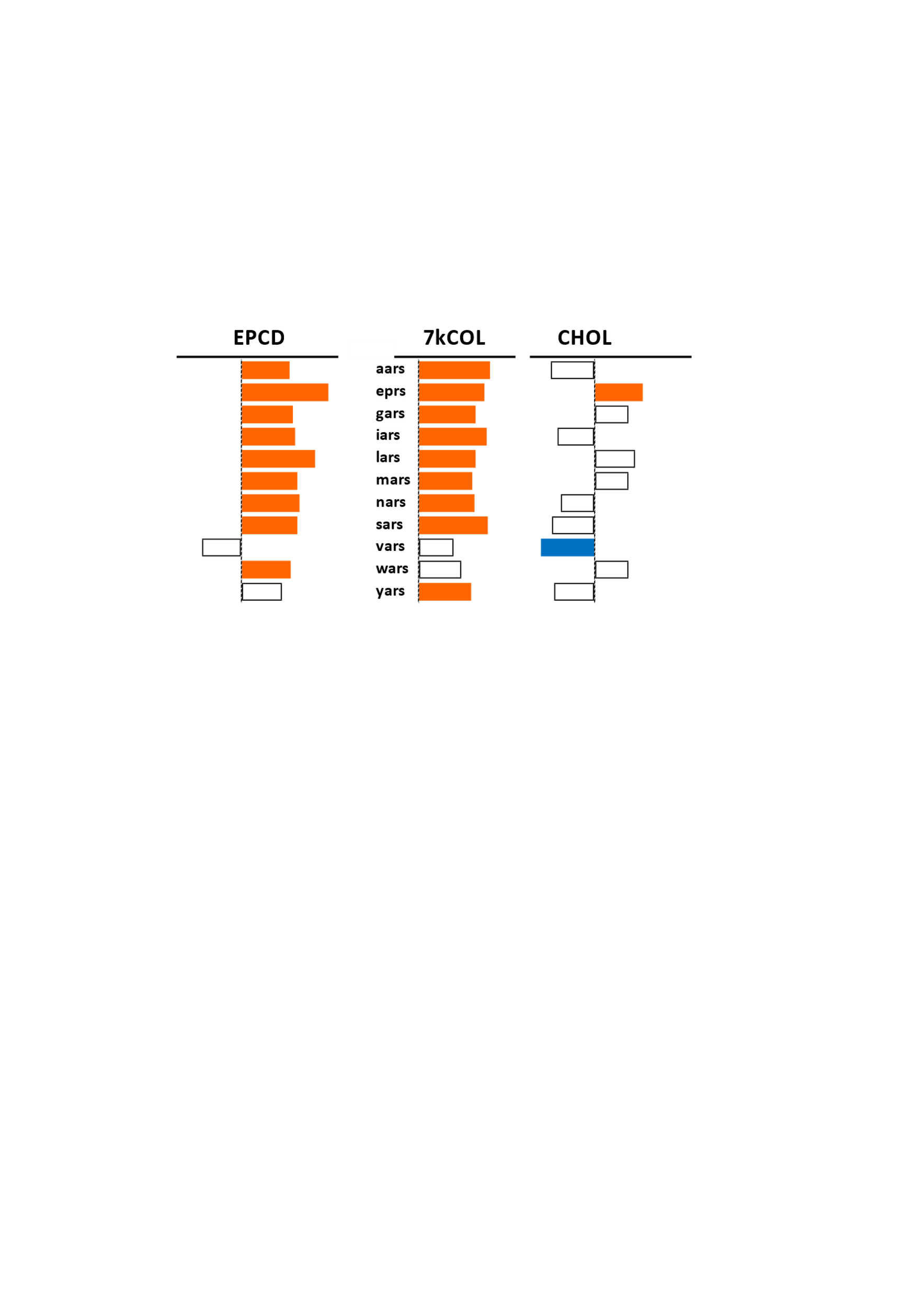


**Figure S6**: Array results for selected genes coding for aminoacyl transfer RNA synthetases.

S.3.1. T antigen, *Trp53*-associated gene expression, and cell cycle arrest in 661W cells

S.3.1.1. Introduction

Classically termed an oncogene, p53 is a transcriptional regulator with a varied set of targets, controlling a wide distribution of processes, including apoptosis, cell cycle, response to reactive oxygen species, and autophagy, as well as constitutive functions [15]. The overall p53 response is flexible and probably attuned to cell type, differentiation state, and specific stress conditions. In non-transformed cells p53 expression and activity increase in response to a variety of challenges, most notably DNA damage, initiating pathways that modulate DNA repair and replication, and therefore ultimately affecting the cell cycle. p53 target genes may be associated with cell cycle arrest, cell death and/or cell survival pathways [16,17]. An additional consideration regarding p53 is that the 661W cell line was immortalized using the SV40 large T antigen [18], which could have an impact on p53-dependent differential gene expression that otherwise would result from responses to the experimental treatments used for this gene array study, particular those that affect cell viability. We therefore extended enrichment analysis to evaluate results related to the p53 signaling pathway (**Figure S7**). A central finding was that *Trp53* gene expression was significantly down-regulated by both EPCD and CHOL, but not 7kCHOL (**Figure S5** (for CHOP-associated genes)), and the following results were interpreted in that context.

S.3.1.2. Gene enrichment involving p53

Multiple forms of stress, the most prominent being DNA damage, but also oxidative and ER stress, are known to promulgate increased activity of p53, as a mediator of events both protecting the affected cell, as well as initiating cell death pathways, depending on the context and nature of the stimulus [19,20]. Unsurprisingly, given their transformation by SV40 T antigen, analysis of gene expression in (oxy)sterol-treated 661W cells related to p53 signaling yielded mixed results (**Figure S7**). For GO: terms based on p53 signaling, and specifically for response to DNA damage within this category, incubation with oxysterols led to statistically significant expression of upregulated genes, with EPCD being the only treatment resulting in a group of down-regulated genes that barely surpassed the *P* ≤ 0.05 threshold for enrichment significance for the term “p53-mediated DNA damage response” (**Figure S7**, Columns A and C) (The sets of oxysterol-induced DEGs for Columns A and C only partly overlapped). In contrast, CHOL treatment generated only genes with negative FC for the two GO: terms queried in **Figure S7** (Columns A and C), indicating minimal changes related to p53 expression, and for Column C in particular, the results were consistent with those for categories related to DNA damage and repair shown in **Figure 14**. The KEGG term “p53 signaling pathway,” by the definition given (https://www.genome.jp/dbget-bin/www_bget?map04115) seems to be more restrictive, and in this category, fewer genes were captured for oxysterol-treated cells (**Figure S7**, Columns A vs. B). In addition, for this KEGG term, down-regulated genes were more numerous than those upregulated for each treatment (**Figure S7, Column B**). The enrichment results depicted in **Figure S7** as a whole indicate important differences for down-regulated DEGs between the EPCD and CHOL treatment response, given their shared decrease in *Trp53* gene expression (**Figure S5**).

S.3.1.3. p53 signaling in 661W cells

p53 signaling in neurons—without constraints imposed by T antigen—occurs in response to various modes of stress and may lead to cell death or cell cycle arrest, encompassing the transcription of multiple genes involved in these pathways [21]. However, under conditions where p53 activity is inhibited, by both transcriptional and post-translational means, alternative p53-independent mechanisms should be considered to underlie changes in gene expression both upstream and downstream from p53.

Miller *et al*. [22] have detected phosphorylated p53 following challenge of 661W cells with a genotoxic agent that is selectively toxic to photoreceptors. This finding was correlated with both cell cycle arrest, increased expression of BAX, and a high proportion of apoptotic cells, indicating that 661W cells can still maintain at least some functional p53 that is available to participate in the DNA damage response and

**Figure S7**: Gene Enrichment analysis using the following terms: A, Signal transduction by p53 class mediator (GO: term); B, p53 signaling pathway (KEGG term); C, DNA damage response, signaling by p53 class mediator.

resulting cell death. Another report showed that SV40 T antigen-immortalized rodent embryonic fibroblasts underwent p53-dependent apoptosis in response to genotoxic stress [23]. It was proposed that newly synthesized p53 protein is rapidly transported to the nucleus before it has an opportunity to associate with and become inactivated by T antigen, leading to at least low-level transactivation of target genes. There is a possibility that T antigen bound-p53 is still capable of non-transcription-dependent, proapoptotic activity under appropriate stimulus conditions [23]. It is also important to point out that SV40 T antigen-transformed cells are not equivalent to p53 knockouts, as stable expression of large T antigen is not achieved in the latter, and wild-type p53 may exert a helper effect for recruitment of additional transcriptional regulators to a p53/T antigen complex [24]. The predominant isoform (termed LIP) of CEBPβ (which is up-regulated during ER stress (**Figure S5**)), has been demonstrated to induce proteasomal degradation of T antigen [25]; in fact, LIP also inhibits T antigen gene *transcription* [26], providing a plausible scenario for oxysterol-induced stress to override the p53-inhibitory function of T antigen in 661W cells. These findings suggest that the significant down-regulation of *Trp53* in EPCD-treated 661W cells presents an important contrast with lack of differential expression of this gene in 7kCHOL-treated cells. Given the possibility that there may in fact be some basal p53 protein expression in 661W cells maintained under conventional conditions [22], that T antigen expression does not affect p53 gene transcription itself, and that some facets of cell stress responses could attenuate T antigen activity, the observed differences with respect to p53 gene expression between EPCD- and CHOL-treated samples, on the one hand, and those from cells treated with 7kCHOL on the other hand, raise the possibility that 661W cells incubated with 7kCHOL may have more abundant functional p53 than those exposed to the other two treatments. Suppression of p53 translation abrogates protection of the genome from reactive oxygen species-induced DNA damage [20], suggesting that down-regulation of *Trp53* expression by EPCD— and also CHOL, even though employed as a non-toxic control— may increase susceptibility to oxidative stress.

**Supplementary References**

1. Zhang, M.; An, C.; Gao, Y.; Leak, R.K.; Chen, J.; Zhang, F. Emerging roles of Nrf2 and phase II antioxidant enzymes in neuroprotection. *Prog. Neurobiol*. **2013**, *100*, 30–47, doi:10.1016/j.pneurobio.2012.09.003.
2. Jaramillo, M.C.; Zhang, D.D. The emerging role of the Nrf2–Keap1 signaling pathway in cancer. *Genes Dev*. **2013**, *27*, 2179–2191, doi:10.1101/gad.225680.113.
3. Cullinan, S.B.; Zhang, D.; Hannink, M.; Arvisais, E.; Kaufman, R.J.; Diehl, J.A. Nrf2 Is a direct PERK substrate and effector of PERK-dependent cell survival. *Mol. Cell. Biol*. **2003**, *23*, 7198–7209, doi:10.1128/mcb.23.20.7198-7209.2003.
4. Granatiero, V.; Konrad, C.; Bredvik, K.; Manfredi, G.; Kawamata, H. Nrf2 signaling links ER oxidative protein folding and calcium homeostasis in health and disease. *Life Sci. Alliance* **2019**, *2*, e201900563, doi:10.26508/lsa.201900563.
5. Awasthi, Y.C.; Yang, Y.; Tiwari, N.K.; Patrick, B.; Sharma, A.; Li, J.; Awasthi, S. Regulation of 4-hydroxynonenal-mediated signaling by glutathione S-transferases. *Free Radic. Biol. Med*. **2004**, *37*, 607–619, doi:10.1016/j.freeradbiomed.2004.05.033.
6. McBean, G.J.; Aslan, M.; Griffiths, H.R.; Torrão, R.C. Thiol redox homeostasis in neurodegenerative disease. *Redox Biol*. **2015**, *5*, 186–194, doi:10.1016/j.redox.2015.04.004.
7. Zucker, S.N.; Fink, E.E.; Bagati, A.; Mannava, S.; Bianchi-Smiraglia, A.; Bogner, P.N.; Wawrzyniak, J.A.; Foley, C.; Leonova, K.I.; Grimm, M.J.; et al. Nrf2 amplifies oxidative stress via induction of Klf9. *Mol. Cell* **2014**, *53*, 916–928, doi:10.1016/j.molcel.2014.01.033.
8. Rhee, S.G.; Kil, I.S. Mitochondrial H_2_O_2_ signaling is controlled by the concerted action of peroxiredoxin III and sulfiredoxin: Linking mitochondrial function to circadian rhythm. *Free Radic. Biol. Med*. **2016**, *99*, 120–127, doi:10.1016/j.freeradbiomed.2016.07.029.
9. Schieber, M.; Chandel, N.S. ROS function in redox signaling and oxidative stress. *Curr. Biol*. **2014**, *24*, R453–462, doi:10.1016/j.cub.2014.03.034.
10. Tebay, L.E.; Robertson, H.; Durant, S.T.; Vitale, S.R.; Penning, T.M.; Dinkova-Kostova, A.T.; Hayes, J.D. Mechanisms of activation of the transcription factor Nrf2 by redox stressors, nutrient cues, and energy status and the pathways through which it attenuates degenerative disease. *Free Radic. Biol. Med*. **2015**, *88*, 108–146, doi:10.1016/j.freeradbiomed.2015.06.021.
11. Valera-Alberni, M.; Cantó, C. Mitochondrial stress management: A dynamic journey. *Cell Stress* **2018**, *2*, 253–274, doi:10.15698/cst2018.10.158.
12. Fugio, L.B.; Coeli-Lacchini, F.B.; Leopoldino, A.M. Sphingolipids and mitochondrial dynamic. *Cells* **2020**, *9*, 581, doi:10.3390/cells9030581.
13. Hernando-Rodríguez, B.; Artal-Sanz, M. Mitochondrial quality control mechanisms and the PHB (prohibitin) complex. *Cells* **2018**, *7*, 238, doi:10.3390/cells7120238.
14. Cummins, N.; Götz, J. Shedding light on mitophagy in neurons: What is the evidence for PINK1/Parkin mitophagy in vivo? *Cell. Mol. Life Sci*. **2018**, *75*, 1151–1162, doi:10.1007/s00018-017-2692-9.
15. Kastenhuber, E.; Lowe, S. Putting p53 in context. *Cell* **2017**, *170*, 1062–1078, doi:10.1016/j.cell.2017.08.028.
16. Fischer, M. Census and evaluation of p53 target genes. *Oncogene* **2017**, *36*, 3943–3956, doi:10.1038/onc.2016.502.
17. Kracikova, M.; Akiri, G.; George, A.; Sachidanandam, R.; Aaronson, S.A. A threshold mechanism mediates p53 cell fate decision between growth arrest and apoptosis. *Cell Death Differ*. **2013**, *20*, 576–588, doi:10.1038/cdd.2012.155.
18. Al-Ubaidi, M.R.; Font, R.L.; Quiambao, A.B.; Keener, M.J.; Liou, G.I.; Overbeek, P.A.; Baehr, W. Bilateral retinal and brain tumors in transgenic mice expressing simian virus 40 large T antigen under control of the human interphotoreceptor retinoid-binding protein promoter. *J. Cell Biol*. **1992**, *119*, 1681–1687, doi:10.1083/jcb.119.6.1681.
19. Lopez, I.; Tournillon, A.S.; Nylander, K.; Fåhraeus, R. p53-mediated control of gene expression via mRNA translation during endoplasmic reticulum stress. *Cell Cycle* **2015**, *14*, 3373–3378, doi:10.1080/15384101.2015.1090066.
20. Martindale, J.L.; Holbrook, N.J. Cellular response to oxidative stress: Signaling for suicide and survival. *J. Cell. Physiol*. **2002**, *192*, 1–15, doi:10.1002/jcp.10119.
21. Culmsee, C.; Mattson, M.P. p53 in neuronal apoptosis. *Biochem. Biophys. Res. Comm*. **2005**, *331*, 761–777, doi:10.1016/j.bbrc.2005.03.149.
22. Miller, T.J.; Schneider, R.J.; Miller, J.A.; Martin, B.P.; Al-Ubaidi, M.R.; Agarwal, N.; Dethloff, L.A.; Philbert, M.A. Photoreceptor cell apoptosis induced by the 2-nitroimidazole radiosensitizer, CI-1010, is mediated by p53-linked activation of caspase-3. *Neurotoxicology* **2006**, *27*, 44–59, doi:10.1016/j.neuro.2005.06.001.
23. Cole, S.L.; Tevethia, M.J. Simian virus 40 large T antigen and two independent T-antigen segments sensitize cells to apoptosis following genotoxic damage. *J. Virol*. **2002**, *76*, 8420–8432, doi:10.1128/JVI.76.16.8420–8432.2002.
24. Hermannstädter, A.; Ziegler, C.; Kühl, M.; Deppert, W.; Tolstonog, G.V. Wild-type p53 enhances efficiency of simian virus 40 large-T-antigen-induced cellular transformation. *J. Virol*. **2009**, *83*, 10106–10118, doi:10.1128/JVI.00174-09.
25. Bellizzi, A.; White, M.K.; Wollebo, H.S. Degradation of polyomavirus JC T-antigen by stress involves the LIP isoform of C/EBPβ. *Cell Cycle* **2015**, *14*, 2075–2079, doi:10.1080/15384101.2015.1042631.
26. Romagnoli, L.; Wollebo, H.S.; Deshmane, S.L.; Mukerjee, R.; Del Valle, L.; Safak, M.; Khalili, K.; White, M.K. Modulation of JC virus transcription by C/EBPbeta. *Virus Res*. **2009**, *146*, 97–106, doi:10.1016/j.virusres.2009.09.005.

**Legends for Supplementary Tables as Excel spreadsheets:**

**Supplementary Tables S1 (A, B):**

**Supplemental Table S1A:** Differentially expressed genes (DEGs) with the 20 highest "-Fold Change" (FC) vs. vehicle control samples for EPCD, 7kCHOL, and CHOL treatments. Also shown are AdjP values. Genes with symbols highlighted in yellow are shared by EPCD and 7kCHOL; those with green highlight are shared between EPCD and CHOL.

**Supplemental Table S1B:** Differentially expressed genes (DEGs) with the 20 lowest "-Fold Change" (FC) vs. vehicle control samples for EPCD, 7kCHOL, and CHOL treatments. Also shown are AdjP values. Genes with symbols highlighted in yellow are shared by EPCD and 7kCHOL; those with green highlight are shared between EPCD and CHOL; Sema7a (highlighted in pink) is in common with all three experimental treatments. Bold outline: DEGs down-regulated by 7kCHOL that are involved in cholesterol synthesis.

**Supplemental Tables S2 (A, B, C):**

Complete lists of genes differentially expressed for each of the three experimental treatments (EPCD, 7kcCHOL, and CHOL. N1.5 and P1.5 denote genes with FC ≤ -1.5 and FC ≥ 1.5, respectively; these lists were used for enrichment analysis and selection of DEGs for charts. For comparison, genes listed under the headings N2 and P2 were DEGs with FC ≤ -2 and FC ≥ 2, respectively. All listed genes also had AdjP ≤ 0.0010.

**Supplementary Table S3: Adjusted P-values for Threshold “-Fold Change”**

**Positive and Negative Values**

^1^Positive Fold Change includes all results with FC ≥ 1.5; Negative, those with FC ≤ -1.5

**Supplementary Table S4: Genes associated with Hmox1 and heme catabolism**

Values represent change in expression vs. VC. Up-regulated DEGs are highlighted in

bolded orange; down-regulated DEGs in bolded blue.

**Supplemental Table S5: Total RNA measurements from gene array samples**

CD: cyclodextrin vehicle control; 7k: 7kCHOL-treated; E: EPCD-treated; Ch: CHOL-treated
